# Variants in mitochondrial amidoxime reducing component 1 and hydroxysteroid 17‐beta dehydrogenase 13 reduce severity of nonalcoholic fatty liver disease in children and suppress fibrotic pathways through distinct mechanisms

**DOI:** 10.1002/hep4.1955

**Published:** 2022-04-11

**Authors:** Christian A. Hudert, Leon A. Adams, Anna Alisi, Quentin M. Anstee, Annalisa Crudele, Laura G. Draijer, EU‐PNAFLD investigators, Samuel Furse, Jan G. Hengstler, Benjamin Jenkins, Kylie Karnebeek, Deirdre A. Kelly, Bart G. Koot, Albert Koulman, David Meierhofer, Phillip E. Melton, Trevor A. Mori, Stuart G. Snowden, Indra van Mourik, Anita Vreugdenhil, Susanna Wiegand, Jake P. Mann

**Affiliations:** ^1^ Department of Pediatric Gastroenterology, Nephrology and Metabolic Diseases Charité Universitätsmedizin Berlin Berlin Germany; ^2^ Medical School University of Western Australia Perth Australia; ^3^ Department of Hepatology Sir Charles Gairdner Hospital Perth Australia; ^4^ Research Unit of Molecular Genetics of Complex Phenotypes Bambino Gesù Children's Hospital‐Istituto di Ricovero e Cura a Carattere Scientifico Rome Italy; ^5^ 5994 Translational and Clinical Research Institute Faculty of Medical Sciences Newcastle University Newcastle upon Tyne UK; ^6^ Newcastle National Institute for Health Research Biomedical Research Centre Newcastle upon Tyne Hospitals National Health Service Foundation Trust Newcastle upon Tyne UK; ^7^ Department of Pediatric Gastroenterology and Nutrition Amsterdam University Medical Center Emma Children’s Hospital University of Amsterdam Amsterdam the Netherlands; ^8^ Core Metabolomics and Lipidomics Laboratory Wellcome Trust–Medical Research Council Institute of Metabolic Science University of Cambridge Cambridge UK; ^9^ Systems Toxicology Leibniz Research Center for Working Environment and Human Factors at the Technical University Dortmund Dortmund Germany; ^10^ Center for Overweight Adolescent and Children's Health Care Department of Pediatrics Maastricht University Medical Center Maastricht the Netherlands; ^11^ Liver Unit Birmingham Womens and Children’s Hospital Trust Birmingham UK; ^12^ Max Planck Institute for Molecular Genetics Mass Spectrometry Facility Berlin Germany; ^13^ School of Global Population Health Faculty of Health and Medical Sciences University of Western Australia Perth Australia; ^14^ School of Pharmacy and Biomedical Sciences Faculty of Health Sciences Curtin University Perth Australia; ^15^ Menzies Institute for Medical Research College of Health and Medicine University of Tasmania Hobart Australia; ^16^ Center for Chronically Sick Children Charité Universitätsmedizin Berlin Berlin Germany; ^17^ 2152 Institute of Metabolic Science University of Cambridge Cambridge UK

## Abstract

Genome‐wide association studies in adults have identified variants in hydroxysteroid 17‐beta dehydrogenase 13 (*HSD17B13*) and mitochondrial amidoxime reducing component 1 (*MTARC1*) as protective against nonalcoholic fatty liver disease (NAFLD). We aimed to test their association with pediatric NAFLD liver histology and investigate their function using metabolomics. A total of 1450 children (729 with NAFLD, 399 with liver histology) were genotyped for rs72613567T>TA in *HSD17B13*, rs2642438G>A in *MTARC1*, and rs738409C>G in patatin‐like phospholipase domain‐containing protein 3 (*PNPLA3*). Genotype–histology associations were tested using ordinal regression. Untargeted hepatic proteomics and plasma lipidomics were performed in a subset of children. We found rs72613567T>TA in *HSD17B13* to be associated with lower odds of NAFLD diagnosis (odds ratio, 0.7; 95% confidence interval, 0.6–0.9) and a lower grade of portal inflammation (*p *< 0.001). rs2642438G>A in *MTARC1* was associated with a lower grade of hepatic steatosis (*p* = 0.02). Proteomics found reduced expression of HSD17B13 in carriers of the protective ‐TA allele. MTARC1 levels were unaffected by genotype. Both variants were associated with down‐regulation of fibrogenic pathways. *HSD17B13* perturbs plasma phosphatidylcholines and triglycerides. *In silico* modeling suggested p.Ala165Thr disrupts the stability and metal binding of *MTARC1*. *Conclusion:* Both *HSD17B13* and *MTARC1* variants are associated with less severe pediatric NAFLD. These results provide further evidence for shared genetic mechanisms between pediatric and adult NAFLD.

## Graphical abstract

Here, we found that a common variant in *HSD17B13* reduced the risk of NAFLD diagnosis in children. Another common variant, in *MTARC1*, reduces the severity of steatosis on biopsy. The variant in *HSD17B13* lowers protein expression, whilst the *MTARC1* variant appears to act by affecting enzyme function.

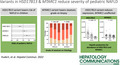

## INTRODUCTION

Understanding genetic variants associated with human chronic disease phenotypes has yielded insights into multifactorial pathogenesis.^[^ ^1^ ^]^ This is of particular importance in nonalcoholic fatty liver disease (NAFLD) as it is a common condition with the potential to progress to end‐stage liver disease and hepatocellular carcinoma^[^ ^2^, ^3^ ^]^ yet has no licensed therapies.^[^ ^4^ ^]^

Population level genome‐ and exome‐wide association studies have identified several common variants implicated in the severity of NAFLD.^[^ ^5^ ^]^ p.Ile148Met in patatin‐like phospholipase domain‐containing protein 3 (*PNPLA3*) is the variant most strongly associated with increased severity of NAFLD^[^ ^6^, ^7^ ^]^ and has been well studied, leading to its identification as a lipid droplet‐binding protein that influences the recruitment of hydrolyzing enzymes.^[^ ^8^, ^9^ ^]^

More recently, strong human genetic evidence has identified two protective variants at genome‐wide significance in adults: rs72613567T>TA in hydroxysteroid 17‐beta dehydrogenase 13 (*HSD17B13*)^[^ ^10^ ^]^ and p.Ala165Thr in mitochondrial amidoxime reducing component 1 (*MTARC1*, rs2642438G>A).^[^ ^11^, ^12^, ^13^, ^14^, ^15^ ^]^

*HSD17B13* has also been implicated in altered lipid metabolism, including recently in regulation of phospholipids^[^ ^16^ ^]^ and retinol.^[^ ^17^ ^]^ However, the function of *MTARC1* in hepatic lipid metabolism is largely unknown, although it clearly has drug detoxifying activity^[^ ^18^, ^19^ ^]^ and has recently been implicated in phospholipid metabolism.^[^ ^20^ ^]^

Histologic validation of genetic variants is challenging due to the comparatively small numbers of patients who undergo liver biopsy for NAFLD. This is even more so in the case of pediatric NAFLD; therefore, analyses study candidate genes in relatively small cohorts.^[^ ^21^, ^22^ ^]^ One genome‐wide association study (GWAS) was conducted in Hispanic boys with biopsy‐proven NAFLD, and this yielded several suggestive loci.^[^ ^23^ ^]^ It is still unclear precisely how closely the genetics of adult and pediatric NAFLD overlap. To date, there is one report of the splice variant in *HSD17B13* being associated with lower alanine aminotransferase (ALT) and a lower proportion of ultrasonographic diagnosis of NAFLD in children.^[^ ^24^ ^]^

Pediatric NAFLD is common (7.6% in the general population^[^ ^25^ ^]^) and shows a predominance of periportal inflammation and zone 1 steatosis, particularly in younger male patients.^[^ ^26^, ^27^ ^]^ While both are positively associated with insulin resistance and other features of the metabolic syndrome,^[^ ^28^ ^]^ it is not clear whether pediatric periportal inflammation in NAFLD is simply a childhood manifestation of adult nonalcoholic steatohepatitis (NASH; with lobular inflammation and ballooning) or a different pathophysiological entity.

Therefore, we sought to address whether the protective variants in *HSD17B13* and *MTARC1* identified at a population level in adults can be replicated in children with NAFLD and to provide histologic data on both variants in children. In this study, we used liver tissue proteomics and plasma lipidomics to gain insight into the impact of these variants on hepatic metabolism in children with NAFLD.

## MATERIALS AND METHODS

### Participants

Two groups of participants were included in this cross‐sectional study, cases (children with NAFLD) and controls (without NAFLD). A subset of children with NAFLD had undergone liver biopsy for clinical indications. All participants (or their parents) gave written informed consent.

Participants were recruited from Cambridge and Birmingham (UK) as part of the European Paediatric NAFLD Registry (EU‐PNAFLD) (Clintrials.gov NCT:04190849),^[^ ^29^ ^]^ which was approved by the East Midlands—Nottingham 2 Research Ethics Committee (17/EM/0084), Maastricht University Medical Center (under ethical approval METC 13‐4‐130), Charité Berlin (under ethical approval of the local institutional review board EA2/049/14),^[^ ^30^ ^]^ Amsterdam University Medical Center (under ethical approval MEC 2017_306 and MEC 07/141), and Bambino Gesu Hospital (under ethical approval for EU‐PNAFLD and local ethics review board, protocol number 1774_OPBG_2019). Children were referred to these clinics due to obesity and/or abnormal liver biochemistry and were then subsequently investigated for comorbidities, including NAFLD. A subset of patients with NAFLD underwent liver biopsy for clinical indications.

Data were also included from the Raine Study, a population‐level cohort study in Western Australia that is described in further detail elsewhere.^[^ ^31^ ^]^ Briefly, adolescents (17 years of age) were invited to participate in a prospective long‐term study with detailed metabolic phenotyping, including an abdominal ultrasound scan for identification of hepatic steatosis (NAFLD). Participants from the Raine Study cohort contributed to controls and NAFLD cases based on the criteria below.

Cases and controls were recruited from the same clinics (and referral populations); this aimed to reduce the bias of case–control comparisons. Recruitment from multiple different hospitals aimed to reduce bias associated with a sample from a single hospital population. As an exploratory analysis, we used data from the maximum number of available participants, and therefore no formal sample size calculation was performed.

### Inclusion and exclusion criteria

All participants were 5–18 years old at the time of inclusion. All cases (n = 729) were identified according to North American Society for Pediatric Gastroenterology, Hepatology, and Nutrition criteria,^[^ ^32^ ^]^ with radiologic evidence of steatosis and/or elevated ALT (>50 U/L for boys, >44 U/L for girls) in subjects who were overweight or obese. Liver biopsy with histologic characterization of NAFLD was available for 399 (55%) NAFLD cases. Controls (n = 721) were those with NAFLD excluded by both absence of hepatic steatosis following radiologic examination and normal ALT.^[^ ^32^ ^]^

Exclusion criteria were age <5 or >18 years, any other liver disease (assessment for secondary causes, including alpha‐1‐antitrypsin deficiency; celiac disease; autoimmune hepatitis; viral hepatitis A, B, and C; active cytomegalovirus or Epstein‐Barr virus infection; thyroid disorders; and Wilson’s disease), severe underlying chronic disease (e.g., cardiopulmonary or autoimmune disease), alcohol consumption >20 g of alcohol per day, and pregnancy.

Children with incomplete data, inadequate genotyping quality, or unclear genotyping calls were also excluded. Forty‐two patients initially recruited were excluded: 32 were under 5 or over 18 years old, five with unclear diagnosis, and five without adequate data.

### Clinical and laboratory investigations

For all participants, anthropometric measures (height, weight) were taken; laboratory analysis, including a hepatic panel and complete blood count, was performed; and fasted‐state metabolic parameters were assessed by using standardized procedures. The homeostatic model assessment of insulin resistance (HOMA‐IR) was derived using fasting insulin (µU/L) × fasting glucose (nmol/L) / 22.5. Overweight was defined as body mass index (BMI) *z* score >1; obesity was defined as BMI *z* score >2.

### Genotyping

For the Raine Study cohort, DNA was extracted from whole blood using a Puregene DNA isolation kit. Genotyping was performed on an Illumina BeadArray Reader with the Illumina Human660‐W Quad Array and imputation using MACH v.1.0.16 against a reference of the North and Western European genetic ancestry samples of HapMap phase 2, build 36, release 22. All variants included in the study passed quality thresholds of Hardy‐Weinberg *p* value >5.7 × 10^7^ and imputation quality >0.8, as used in the original description of this cohort.^[^ ^31^ ^]^

For all other participants, DNA was extracted from whole blood (using Qiagen DNeasy kit #69504). All participants were genotyped by quantitative polymerase chain reaction using the following TaqMan assays (Thermo Fisher #4351379): rs738409C>G in *PNPLA3*, rs2642438G>A in *MTARC1*, and rs72613567T>TA in *HSD17B13* (using the custom sequence from Pirola et al.^[^ ^33^ ^]^). These variants were selected due to their evidence as genome‐wide risk factors for NAFLD and cirrhosis in adults.^[^ ^6^, ^7^, ^10^, ^11^, ^34^, ^35^, ^36^ ^]^ Data on variants in *HSD17B13, MTARC1, and PNPLA3* were available for 1412, 1358, and 1165 participants, respectively. Hardy‐Weinberg equilibrium in the control groups using chi‐squared tests were rs2642438G>A in *MTARC1*, *Q* = 0.53; rs72613567T>TA in *HSD17B13*, *Q* = 0.36; and rs738409C>G in *PNPLA3*, *Q* = 0.06.

### Liver biopsies

Liver biopsies were evaluated and scored by experienced pathologists from the respective centers. Staging and grading were performed according to the histologic scoring system for NAFLD by the NASH‐Clinical Research Network (NASH‐CRN).^[^ ^37^ ^]^ Briefly, grading included the scoring of steatosis (0, <5%; 1, 5%–33%; 2, 34%–66%; 3, ≥67%), lobular inflammation (0, 0 foci/200× field; 1, <2 foci/200× field; 2, 2–4 foci/200× field; 3, >4 foci/ 200× field), and hepatocellular ballooning (0, none; 1, few; 2, many/prominent). Portal inflammation was evaluated according to Brunt et al.^[^ ^38^ ^]^ (0, none; 1, mild; 2, moderate to severe). Staging of fibrosis was performed using NASH‐CRN criteria (0, no fibrosis; 1, zone 3 perisinusoidal only or portal/periportal without bridging only; 2, zone 3 perisinusoidal + portal/periportal; 3, bridging fibrosis; 4, cirrhosis).

### Hepatic proteomic analysis

Hepatic tissue proteomics was performed in a subset of 70 patients, as described.^[^ ^30^ ^]^ Liver biopsy specimens were extracted under denaturing conditions and digested by trypsin for subsequent analysis by mass spectrometry.^[^ ^39^ ^]^ The software tools MaxQuant^[^ ^40^ ^]^ and gene set enrichment analysis (GSEA)^[^ ^41^ ^]^ were used for peptide identification and pathway analyses, respectively (see Supporting Methods for details).

### *In silico* analysis of MTARC1 p.Ala165Thr

Our hepatic proteomics data suggested that this *MTARC1* variant did not alter expression of MTARC1 protein, unlike the studied *HSD17B13* variant. Therefore, to further provide some insight into the impact of rs2642438G>A (p.Ala165Thr) in *MTARC1*, we used a range of bioinformatics tools to perform an *in silico* analysis of the variant. UniProt^[^ ^42^ ^]^ was searched for isoforms of *MTARC1* in other species, and sequences were aligned. Four tools were chosen to be used for prediction of the impact of the missense variant based on recommendations of accuracy from the range of tools available^[^ ^43^, ^44^ ^]^: SNPs&GO,^[^ ^45^ ^]^ PANTHER,^[^ ^46^ ^]^ Align‐GVGD,^[^ ^47^ ^]^ and MutPred2.^[^ ^48^ ^]^ For a structural analysis, we used the available crystal structure of MTARC1^[^ ^49^ ^]^ and three tools for calculating the effect of p.Ala165Thr on overall protein stability: I‐Mutant3.0,^[^ ^50^ ^]^ DUET,^[^ ^51^ ^]^ and CUPSAT.^[^ ^52^ ^]^ Data on *in silico* saturation mutagenesis of MTARC1 were available from EVmutation. An annotated protein model was generated using University of California San Francisco Chimera.^[^ ^53^ ^]^

### Plasma lipidomics analysis

Plasma lipidomics was performed in a subset of 141 children with NAFLD. The methods have been described in detail elsewhere^[^ ^54^ ^]^ and in the Supporting Methods. In brief, fasting plasma samples were analyzed by liquid chromatography with mass spectrometry detection. Full chromatographic separation of intact lipids was achieved using the Shimadzu high‐performance liquid chromatography system (Shimadzu UK Limited, Milton Keynes, UK) with the injection of 10 µL onto a Waters Acquity Ultra Performance Liquid Chromatography Charged Surface Hybrid C18 column (Waters, Hertfordshire, UK). The mass spectrometer used was the Thermo Scientific Exactive Orbitrap with a heated electrospray ionization source (Thermo Fisher Scientific, Hemel Hempstead, UK).

For data processing, responses of analytes were normalized to the relevant internal standard response, which were then blank corrected. Accepted area ratios were multiplied by the concentration of the internal standard to give the analyte semiquantitative concentrations. Only lipid species detected in >70% of participants (including lean controls) were included. For included species, minimum value imputation was used for missing values.

### Statistical analyses

Testing for normal distribution was performed for all variables using the Shapiro‐Wilk method. Frequencies and percentages are presented for clinical (categorical) and histologic (ordinal) characteristics. Medians and quartiles of continuous anthropometric and laboratory parameters were calculated for the total study population as well as for the following subgroups: presence/absence of NAFLD, variant genotype within cases, and patients with proteomic or lipidomic profiles.

We used the Kruskal‐Wallis test for continuous non‐normally distributed values (age, BMI *z* score) to evaluate differences in distribution between cases and controls as well as within genotypes of variants. Chi‐squared tests were applied to categorical and ordinal variables (sex and presence of obesity). For all continuous laboratory values, linear regression models with correction for age and sex were used.

Genotype was coded by the number of “protective” alleles: *HSD17B13* rs72613567 T/T=0, T/TA=1, TA/TA=2; and *MTARC1* rs2642438 G/G=0, G/A=1, A/A=2. Hardy‐Weinberg equilibrium was tested using a chi‐squared test with one degree of freedom for all genetic variants.

Case–control analysis for the presence/absence of NAFLD was performed using chi‐squared tests and logistic regression models for distinct genetic modes of inheritance (no particular genetic model [genotypes], additive [trend], and dominant, recessive, or multiplicative [alleles]). Additive genetic models were used in the identification of these variants as significant risk loci,^[^ ^10^, ^11^ ^]^ and therefore we have primarily used the additive model in all analyses. Effects of variants were calculated using Wald tests, odds ratios (ORs), and 95% confidence intervals (95% CIs). False discovery rate‐adjusted *Q* values were calculated using the Benjamini‐Hochberg procedure to adjust for multiple testing. All case–control analyses were adjusted for age, sex, and BMI *z* score.

Associations between genotypes and histologic features were tested using univariate ordinal regression or multivariate ordinal regression models with correction for age and sex. Dichotomous histologic associations were tested by binary logistic regression with correction for age and sex. ORs and 95% CIs were calculated.

For proteomic studies, information is in the Supporting Methods.

For lipidomic studies, absolute abundances for each lipid were logarithmically transformed and standardized (to mean, 0; SD, 1). Logistic regression analyses adjusted for sex and age were run to test for associations between lipid species and genotype (wild‐type vs. heterozygote/homozygote). This strategy was used due to low numbers of TA–TA homozygotes (n = 3 for *HSD17B13*) and A–A homozygotes (n = 9 for *MTARC1*). The beta regression coefficients were then plotted against length of carbon chain and number of double bonds. Due to the high correlation between lipid species, the critical *p* value for significance was defined by 0.05 / √n, where n is the number of identified species from each analytical method (n = 229 lipids, therefore *p *< 3.3** × **10^−3^ was determined as statistically significant). We then performed meta‐regression to examine for trends in lipid saturation or carbon chain length within classes of lipids. Beta regression coefficients from the above models were regressed against double bonds or carbons within lipid classes. *p* values were converted to *Q* values by adjusting for multiple testing using the Benjamini‐Hochberg method.

Statistical analysis was performed in SPSS (SPSS Statistics for Windows, version 25.0; IBM, Armonk, NY). Further regression analysis was performed in Stata v16.1 (StataCorp), and random forest analysis was performed using R 4.0.2.^[^ ^55^ ^]^ In addition, graphs were produced in GraphPad v8.0 for Mac (GraphPad Software, La Jolla, CA).

## RESULTS

### Characteristics of the study population

A total of 1450 children (729 with NAFLD and 721 controls) were included in the study. Children with NAFLD were younger, more likely to be the male sex, and more likely to be obese and more insulin resistant and dyslipidemic (Table 1). They exhibited higher liver transaminases, with median ALT being elevated more than 2 times compared to the control group.

**TABLE 1:** Clinical and laboratory characteristics

Variable	Control (n = 721)	NAFLD (n = 729)	*p* value^a^	*Q* value^a^
Age (years)	16.9 (15.4–17.1)	14.0 (12.0–16.9)	3.8 × 10^−39^	1.3 × 10^−38^
Male sex, n (%)	352 (48.8)	417 (57.2)	0.001	0.001
BMI *z* score	1.0 (0.5–2.5)	2.2 (1.6–2.8)	7.7 × 10^−37^	2.2 × 10^−36^
ALT (U/L)	20 (15–26)	43 (25–73)	1.5 × 10^−90^	2.1 × 10^−89^
AST (U/L)	24 (20–29)	34 (24–48)	3.0 × 10^−52^	2.1 × 10^−51^
GGT (U/L)	14 (11–18)	22 (14–33)	4.7 × 10^−42^	2.2 × 10^−41^
Cholesterol (mg/dl)	155 (138–177)	160 (139–185)	0.040	0.043
LDL (mg/dl)	89 (73–108)	97 (79–112)	8.3 × 10^−5^	1.3 × 10^−4^
HDL (mg/dl)	48 (41–56)	43 (38–50)	3.2 × 10^−14^	6.4 × 10^−14^
Triglycerides (mg/dl)	80 (62–107)	98 (70–140)	1.9 × 10^−12^	3.3 × 10^−12^
HOMA	1.8 (1.2–2.9)	3.4 (2.2–5.2)	1.5 × 10^−20^	3.5 × 10^−20^
*HSD17B13* genotype, n (%)				
TT/TTA/TATA	358 (52.4)/282 (41.3)/43 (6.3)	477 (65.4)/219 (30.0)/33 (4.5)	4.4 × 10^−4^	6.2 × 10^−4^
*MTARC1* genotype, n (%)				
GG/GA/AA	362 (56.1)/238 (36.9)/45 (7.0)	385 (54.0)/280 (39.3)/48 (6.7)	0.309	0.309
*PNPLA3* genotype, n (%)				
CC/CG/GG	332 (59.0)/187 (33.2)/44 (7.8)	260 (43.2)/236 (39.2)/106 (17.6)	0.008	0.009

Note

Data represent frequencies (%) or median (interquartile range) as appropriate. For clinical characteristics, *p* values were calculated using the Mann‐Whitney U test for continuous traits and the chi‐squared test for categorical traits. For plasma markers, *p* values were calculated using linear regression with correction for age and sex. For genotypes, *p* values were calculated using binary logistic regression with correction for age, sex, and BMI *z* score. False discovery rate correction (*Q* value) for multiple comparisons was calculated using the Benjamini‐Hochberg method.

Abbreviations: ALT, alanine aminotransferase; AST, aspartate aminotransferase; BMI, body mass index; GGT, gamma‐glutamyltransferase; HDL, high‐density lipoprotein; HOMA, homeostatic model assessment of insulin resistance; HSD17B13, hydroxysteroid 17‐beta dehydrogenase 13; LDL, low‐density lipoprotein; MTARC1, mitochondrial amidoxime reducing component 1; NAFLD, nonalcoholic fatty liver disease; PNPLA3, patatin‐like phospholipase domain‐containing protein 3.

^a^ Values <0.05 are considered significant.

### Association between variants in *MTARC1, HSD17B13, PNPLA3*, and NAFLD

**FIGURE 1:**
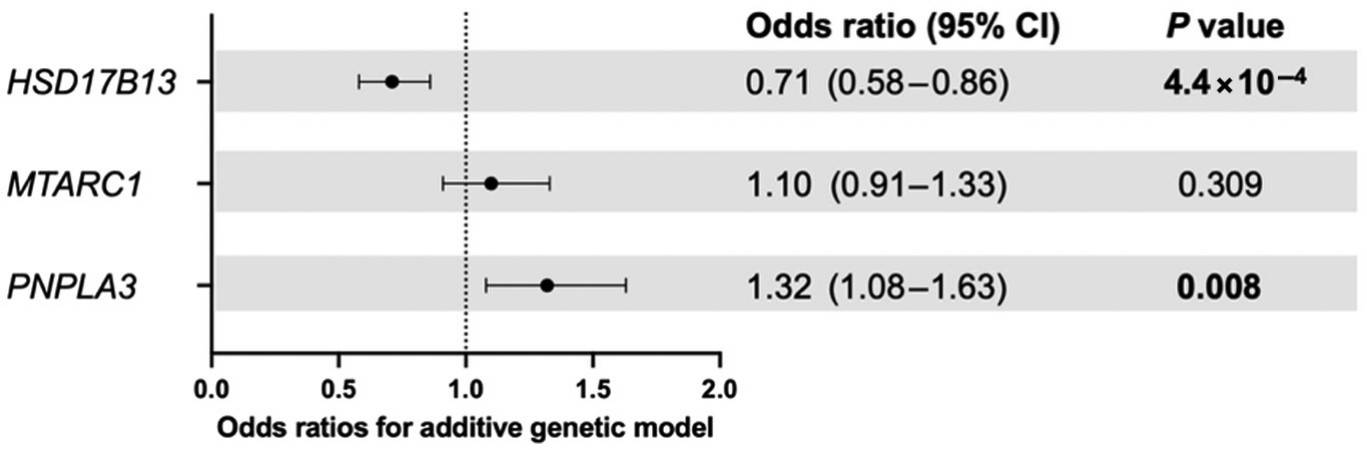
Odds ratios for the presence of NAFLD, using an additive genetic model. *p* values were calculated using logistic regression with correction for age, sex, and BMI *z* score. *PNPLA3* refers to rs738409C>G, *MTARC1* refers to rs2642438G>A, and *HSD17B13* refers to rs72613567T>TA. *p* values in bold denote a significant difference. BMI, body mass index; CI, confidence interval; HSD17B13, hydroxysteroid 17‐beta dehydrogenase 13; MTARC1, mitochondrial amidoxime reducing component 1; NAFLD, nonalcoholic fatty liver disease; PNPLA3, patatin‐like phospholipase domain‐containing protein 3

First, we sought to determine whether these variants were associated with the diagnosis of NAFLD in children (Figure 1). rs738409C>G in *PNPLA3* was positively associated with the diagnosis of NAFLD (OR, 1.32; 95% CI, 1.08–1.63). rs72613567T>TA in *HSD17B13* was protective against the diagnosis of NAFLD (OR, 0.71; 95% CI, 0.58–0.86), and no association with the diagnosis of NAFLD was observed with rs2642438G>A in *MTARC1* (Table S1).

The protective effect of rs72613567T>TA in *HSD17B13* on the diagnosis of NAFLD remained after adjusting for the presence of rs738409C>G in *PNPLA3*, in addition to age, sex, and BMI *z* score (OR, 0.74; 95% CI, 0.57–0.95). The protective effect also remained after adjusting for HOMA‐IR (as a proxy for insulin resistance) (OR, 0.75; 95% CI, 0.61–0.93).

Carriers of the heterozygous or homozygous rs72613567T>TA in the *HSD17B13* variant were slightly older than carriers of the wild‐type variant (16.9 vs. 16.0 years) and had significantly lower transaminases (ALT, 24 vs. 29 IU/L; *Q* = 3.9** × **10^−5^; Table S2). There were no differences in anthropometric or biochemical traits when stratifying all participants by rs2642438G>A in *MTARC1* (Table S3).

### Effect of single nucleotide variants on histologic severity of NAFLD

Of those with NAFLD, 399 (55%) had undergone liver biopsy (Table 2; Table S4). Participants displayed the whole spectrum of NAFLD from simple steatosis to NASH‐associated cirrhosis; 70% had evidence of periportal inflammation, and 14% had advanced fibrosis (stage 3–4).

**TABLE 2:** Histologic findings

Grade/Stage	Steatosis (n = 399)	Portal Inflammation (n = 391)	Lobular Inflammation (n = 399)	Ballooning (n = 393)	Fibrosis (n = 398)
0	–	116 (29.7)	78 (19.5)	124 (31.6)	90 (22.6)
1	91 (22.8)	229 (58.6)	206 (51.6)	166 (42.2)	155 (38.9)
2	186 (46.6)	46 (11.8)	115 (28.8)	103 (26.2)	99 (24.9)
3	122 (30.6)	–	–	–	52 (13.1)
4	–	–	–	–	2 (00.5)

Note

Data represent frequencies (%) of histologic findings in the biopsied study population (n = 399).

**FIGURE 2:**
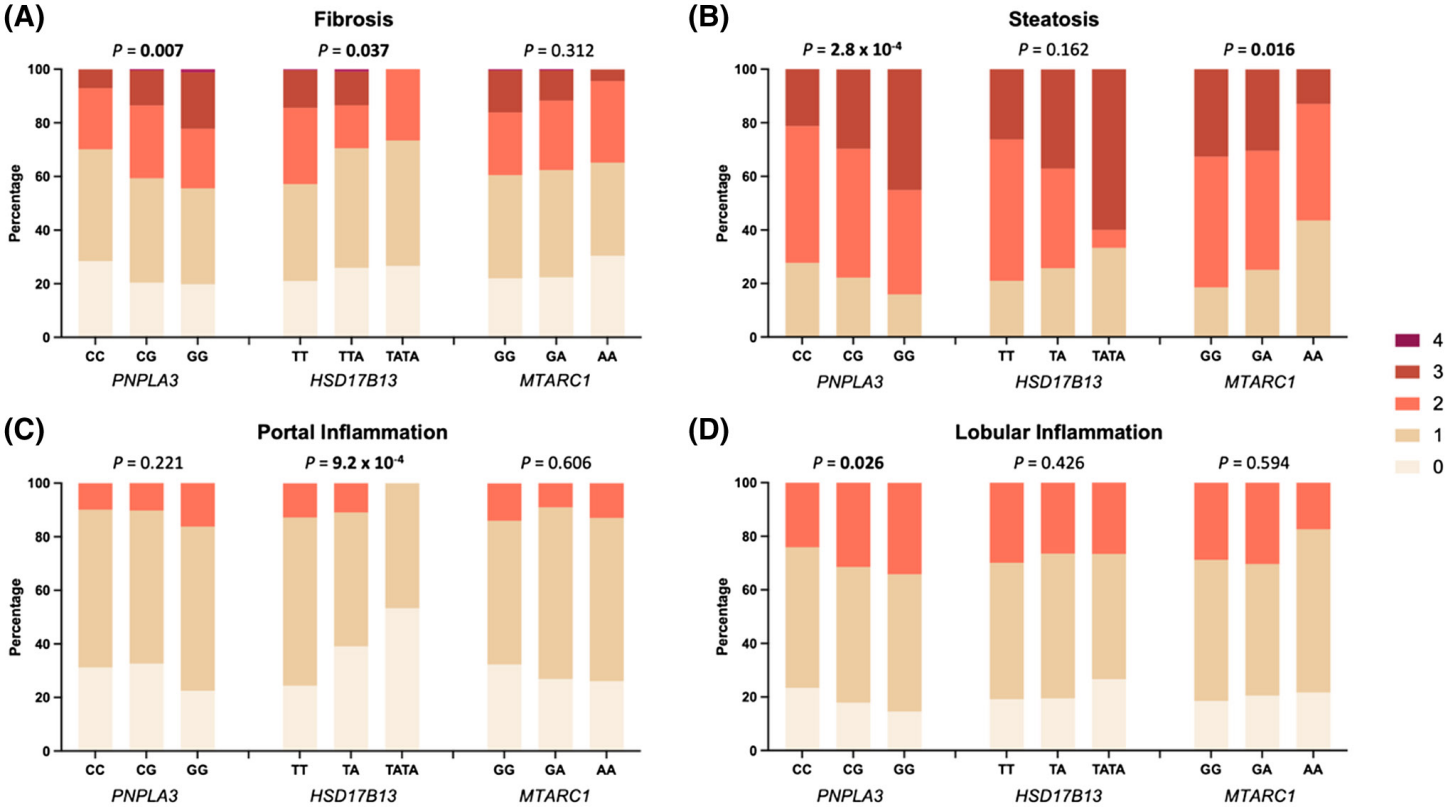
Effect of single nucleotide variants on histologic severity of NAFLD in children. Data from 406 children genotyped for rs738409C>G (*PNPLA3*), rs72613567T>TA (*HSD17B13*), and rs2642438G>A (*MTARC1*). Multivariate ordinal regression analysis with correction for age and sex was performed for the association of the respective genotypes with (A) fibrosis stage (0–4), (B) steatosis grade (1–3), (C) portal inflammation (0–2), and (D) lobular inflammation (0–2). *p* values in bold denote a significant difference. HSD17B13, hydroxysteroid 17‐beta dehydrogenase 13; MTARC1, mitochondrial amidoxime reducing component 1; NAFLD, nonalcoholic fatty liver disease; PNPLA3, patatin‐like phospholipase domain‐containing protein 3

Consistent with its well‐established harmful effect on NAFLD in adults, rs738409C>G in *PNPLA3* was associated with a higher grade of steatosis (*p* = 2.8** × **10^−4^), lobular inflammation (*p* = 0.026), and fibrosis stage (*p* = 0.007) on multivariable ordinal regression adjusted for age and sex (Figure 2; Table S5).

In contrast, variants in *HSD17B13* and in *MTARC1* were associated with less advanced histologic features (Figure 2; Table S5). rs72613567T>TA in *HSD17B13* was strongly associated with a lower grade of portal inflammation (*p* = 9.23** × **10^−4^) as well as a lower stage of fibrosis (*p* = 0.037). rs2642438G>A in *MTARC1* was associated with a lower grade of steatosis (*p* = 0.016).

Similar results were observed using a dichotomous analysis. *PNPLA3* increased odds for the development of both moderate fibrosis (OR, 1.38; 95% CI, 1.03–1.28) and advanced fibrosis (OR, 1.67; 95% CI, 1.07–2.62), while *HSD17B13* was associated with lower odds for moderate fibrosis (OR, 0.61; 95% CI, 0.41–0.91) (Figure S1; Table S6).

### Liver proteomics implicates variants in *HSD17B13* and *MTARC1* in fibrosis and lipid metabolism

**FIGURE 3:**
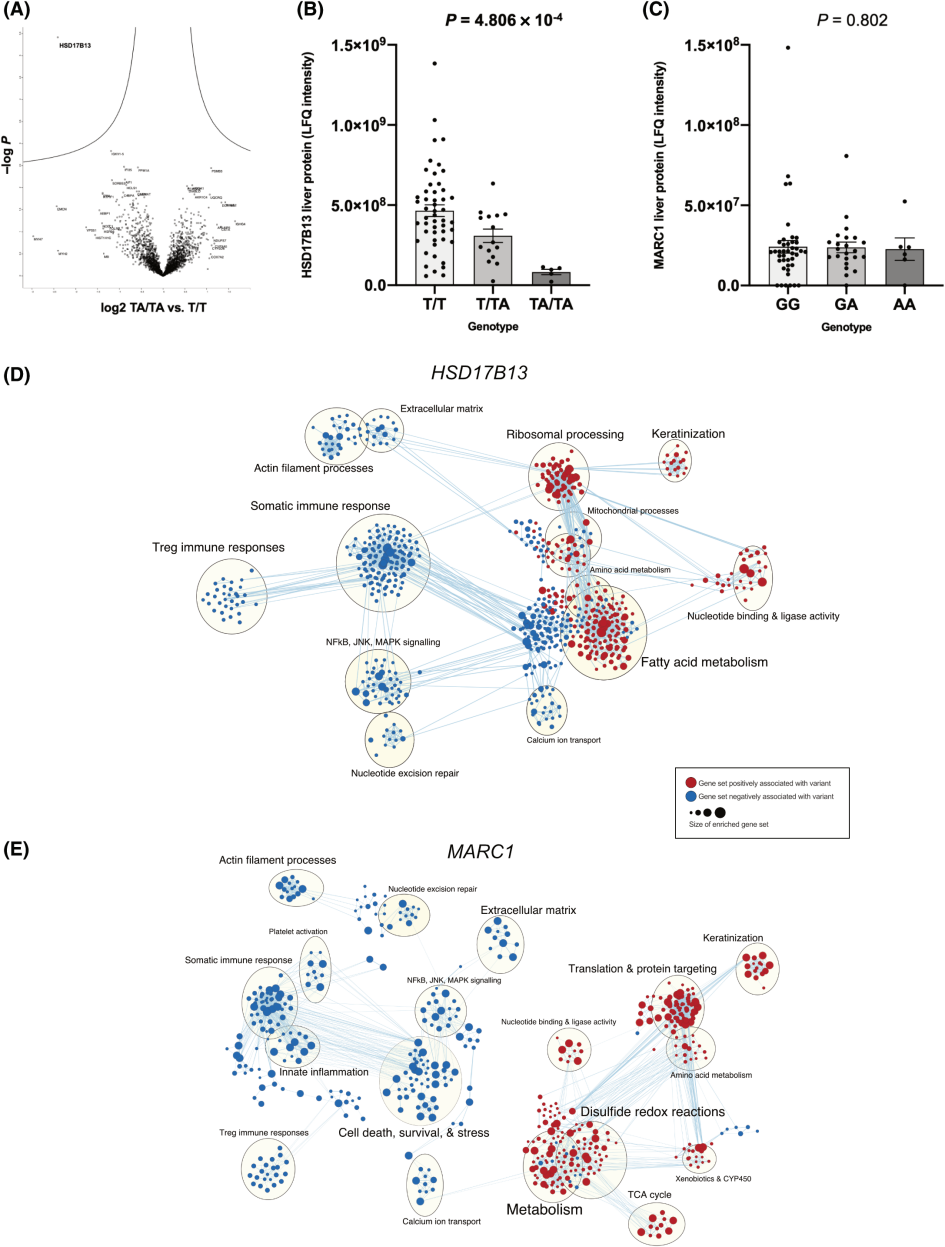
Liver proteomics in children, stratified by rs72613567T>TA in *HSD17B13* and rs2642438G>A in *MTARC1* variants. (A) Volcano plot of log_2_ abundance ratios against the −log_10_ (*p* value) of the proteome for the *HSD17B13* TA/TA versus T/T genotype. (B) Absolute abundance levels (LFQ intensity) stratified by genotype for HSD17B13 liver protein and (C) MTARC1 liver protein. Reported *p* values were calculated using the Kruskal‐Wallis test. (D) Enrichment map of up‐ (red) or down‐regulated (blue) pathways for *HSD17B13* genotype as discovered by gene set enrichment analysis using an additive genetic model. (E) Enrichment map of up‐ (red) or down‐regulated (blue) pathways for *MTARC1* genotype as discovered by gene set enrichment analysis using an additive genetic model. *p* values in bold denote a significant difference. HSD17B13, hydroxysteroid 17‐beta dehydrogenase 13; JNK, c‐Jun N‐terminal kinases; LFQ, label‐free quantitation; MAPK, mitogen‐activated protein kinase; MTARC1, mitochondrial amidoxime reducing component 1; NFkB, Nuclear factor kappa B; TCA, tricarboxylic acid cycle; Treg, T regulatory

To understand the effect of these variants on liver function, we performed proteomics on liver biopsy samples from 70 children with NAFLD who were representative of the overall NAFLD group (Table S7). rs72613567T>TA was associated with lower abundance of HSD17B13 liver protein (Figure 3A,B; Table S8), whereas hepatic levels of MTARC1 protein did not appear to be affected by the rs2642438G>A genotype (Figure 3C; Table S8).

GSEA for rs72613567T>TA in the *HSD17B13* genotype implicated changes in multiple gene sets, including a strong up‐regulation of ribosomal activity (e.g., KEGG_RIBOSOME, normalized enrichment score [NES], 3.0; *Q* = 0) and nonsense‐mediated decay (e.g., REACTOME_NONSENSE_MEDIATED_DECAY, NES, 2.74; *Q* = 0), consistent with degradation of mutant *HSD17B13* (Table S9). When the enriched gene sets were mapped for similarity (Figure 3D), several trends could be observed. There was a strong signature of down‐regulation of pathways associated with immune response (e.g., HALLMARK_INTERFERON_GAMMA_RESPONSE, NES, −2.4; *Q* = 0). Multiple metabolic pathways appeared to be perturbed, including up‐regulation of fatty acid processing (e.g., KEGG_FATTY_ACID_METABOLISM, NES, 2.82; *Q* = 0). There was also down‐regulation of pathways and proteins associated with extracellular matrix formation (e.g., GO_COLLAGEN_CONTAINING_EXTRACELLULAR_MATRIX, NES, −1.9; *Q* = 0.047).

GSEA for rs2642438G>A in *MTARC1* similarly found a strong signature for down‐regulation of extracellular matrix and collagen‐related pathways (e.g., GO_COLLAGEN_TRIMER, NES, −2.46; *Q* = 0.010; and GO_EXTRACELLULAR_MATRIX_STRUCTURAL_CONSTITUENT, NES, −2.35; *Q* = 0.018) as well as gene sets related to the innate immune response (Figure 3E; Table S9). There was strong enrichment in gene sets associated with mitochondrial metabolism, drug detoxification, sphingolipid metabolism, and redox reactions (e.g., GO_SPHINGOLIPID_METABOLIC_PROCESS, NES, 2.15; *Q* = 0.05; and GO_OXIDOREDUCTASE_ACTIVITY, NES, 1.9; *Q* = 0.16).

GSEA for both protective variants demonstrated an up‐regulation of retinol metabolism (NES, 2.45; *Q* = 4.5** × **10^−5^ for *HSD17B13*; NES, 2.14; *Q* = 0.03 for *MTARC1*).

### *In silico* analysis of MTARC1 p.Ala165Thr indicates loss of stability

rs72613567T>TA in *HSD17B13* falls at a splice site, and our proteomics data suggest this results in reduced expression through nonsense‐mediated decay. However, the effect of rs2642438G>A (coding for p.Ala165Thr) on *MTARC1* is less clear as our proteomics results did not show any change in expression of MTARC1 with genotype.

**FIGURE 4:**
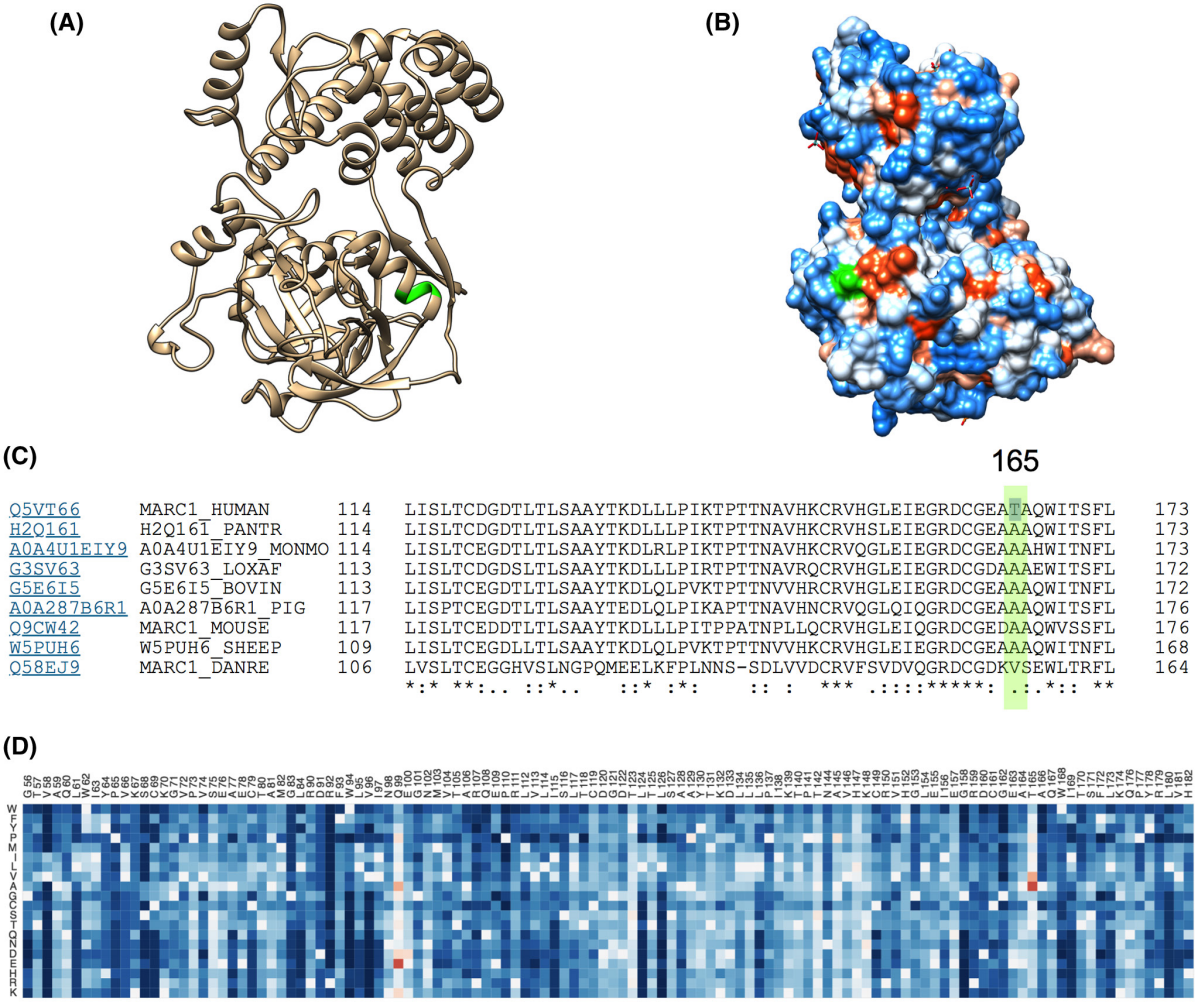
*In silico* prediction of the effects of p.Ala165Thr on *MTARC1*. Alanine‐165 (green) forms (A) part of an alpha helix and (B) part of the external surface of *MTARC1*. (C) This position is highly conserved in mammals, using alignment of protein isoforms. (D) Results of saturation mutagenesis from EVmutation predicts an increase in protein function (red) when substituting alanine for threonine at position 165. MTARC1, mitochondrial amidoxime reducing component 1

*MTARC1* position 165 lies within the cytoplasm (with positions 2–20 within the mitochondrial matrix) and is part of the MOCO sulfurase C‐terminal (MOSC) domain. The crystalline structure of *MTARC1* has been resolved to 1.78Å and shows that alanine‐165 forms part of an alpha‐helix on the external surface of the enzyme (Figure 4A,B). Alanine‐165 is highly conserved across mammals (Figure 4C), although the zebrafish isoform of MTARC1 has a different structure in this region. Using *in silico* saturation mutagenesis, we observed that alanine‐165 is considered to have a substantial beneficial effect on the protein compared to other predicted missense variants in *MTARC1* (Figure 4D). While position 165 is not within any of the predicted active sites of *MTARC1*, two prediction tools classified the p.Ala165Thr variant as disease causing, suggesting that it would cause loss of the alpha‐helix and alter the metal‐binding ability of *MTARC1* (Table S10). Consistent with this, the p.Ala165Thr variant is also predicted to affect the overall stability of the protein.

### Plasma lipidomics shows rs72613567T>TA in *HSD17B13* perturbs triglyceride and phospholipid metabolism

Given that proteomics data implicate these protective variants in lipid metabolism, we performed untargeted plasma lipidomics in 141 children with biopsy‐defined NAFLD who were representative of the overall NAFLD group (Table S11). We tested for associations between variant genotypes and lipid species using logistic regression, adjusted for age and sex.

**FIGURE 5:**
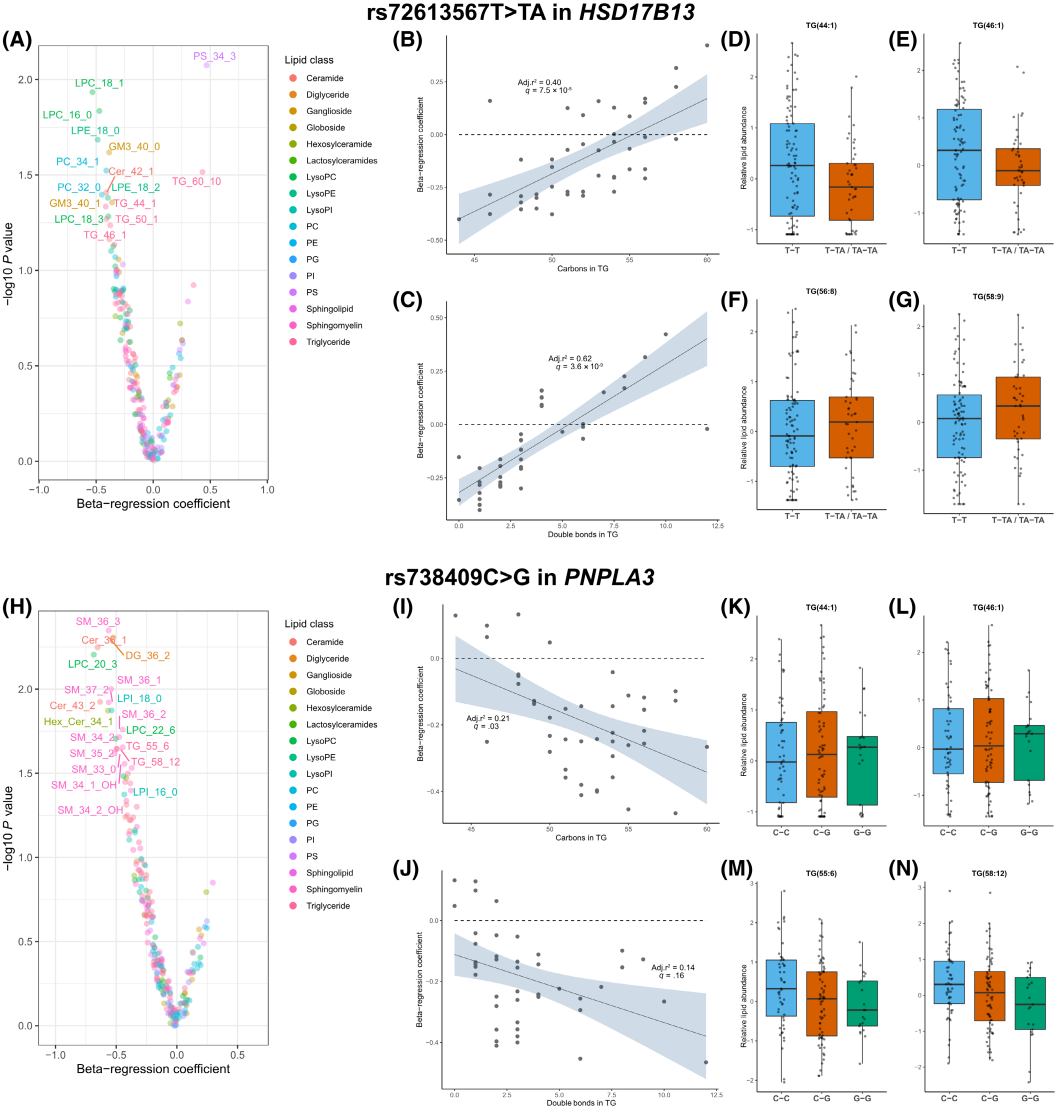
Plasma lipid species associated with rs72613567T>TA in *HSD17B13* and rs738409C>G in *PNPLA3*. (A) Volcano plots demonstrating the association (as beta‐regression coefficient) between lipid species and rs72613567T>TA in HSD17B13, where the beta‐regression coefficient was calculated by logistic regression between genotype (coding T‐T=0, T‐TA/TA‐TA=1) and logarithmically transformed lipid abundance, adjusted for age and sex. rs72613567T>TA in *HSD17B13* was positively associated with (B) TG chain length and (C) saturation, where each data point represents a different TG species. (D–G) Box plots illustrating differences for individual lipids, where each data point represents a child with NAFLD. (H) Volcano plots demonstrating the association (as beta‐regression coefficient) between lipid species and rs738409C>G in PNPLA3, where the beta‐regression coefficient was calculated by logistic regression between genotype (coding T‐T=0, T‐TA/TA‐TA=1) and logarithmically transformed lipid abundance, adjusted for age and sex. (I) rs738409C>G in *PNPLA3* was negatively associated with TG chain length. (K–N) Box plots illustrating differences for individual lipids. Data from 141 children with NAFLD. Cer, ceramide; DG, diglyceride; GM, ganglioside; HSD17B13, hydroxysteroid 17‐beta dehydrogenase 13; LPC, lysophosphatidylcholine; PA, phosphatidic acid; PC, phosphatidylcholine; PE, phosphatidylethanolamine; PG, phosphatidylglycerol; PI, phosphatidylinositol; PNPLA3, patatin‐like phospholipase domain‐containing protein 3; PS, phosphatidylserine; SM, sphingomyelin; TG, triglyceride

rs72613567T>TA in *HSD17B13* was negatively associated with changes in lysophosphatidylcholines, lysophosphatidylethanolamines, and triglycerides (TG) (Figure 5A). This variant was negatively associated with medium‐chain monosaturated TG (e.g., TG[44:1]) and positively associated with very long‐chain polyunsaturated TG (e.g., TG[58:9]; Figure 5B–G). The opposite trend was observed for rs738409C>G in *PNPLA3*, which was negatively associated with very long‐chain polyunsaturated TG (Figure 5I–N). The variant in PNPLA3 was also negatively associated with several sphingomyelin species (Figure 5H).

We also observed that rs72613567T>TA in *HSD17B13* was negatively associated with short‐chain unsaturated phosphatidylcholine (PC) (e.g., PC[28:0]) and positively associated with long‐chain polyunsaturated PC (e.g., PC[37:4]; Figure S2). No significant genotype–lipid associations were observed for rs2642438G>A in *MTARC1* (Tables S12 and S13).

## DISCUSSION

We have provided histologic validation of protective *MTARC1* and *HSD17B13* variants in children with NAFLD. Unlike rs72613567T>TA in *HSD17B13*, hepatic expression of MTARC1 protein is not affected by p.Ala165Thr, suggesting a mechanism of reduced function. Lipidomics analysis found the *HSD17B13* variant to perturb TG and PC metabolism in a contrasting direction to the harmful variant in *PNPLA3*.

Both rs72613567T>TA in *HSD17B13* ^[^ ^10^ ^]^ and rs2642438G>A in *MTARC1* ^[^ ^11^ ^]^ were originally identified as GWAS‐significant risk‐reducing loci for liver disease in adults. This has subsequently been replicated in multiple genome‐wide analyses that also implicate these variants in levels of serum aminotransferases and liver disease in adults.^[^ ^12^, ^13^, ^14^ ^]^ *HSD17B13* has subsequently been validated in multiple cohorts of adults,^[^ ^6^, ^16^, ^17^, ^33^, ^56^, ^57^, ^58^ ^]^ and although a small group of children were included in the original replication cohort where this variant was identified,^[^ ^10^ ^]^ features specific to pediatric NAFLD histology were not described in detail. Similarly, the histologic features associated with the variant in *MTARC1* had not been described in children until now.^[^ ^20^ ^]^ We therefore selected these two variants to study in children in addition to the well‐established risk‐increasing locus rs738409C>G in *PNPLA3*. Here, we observed a protective effect of rs72613567T>TA in *HSD17B13* on the diagnosis of NAFLD in children, which is consistent with the report from Di Sessa et al.^[^ ^24^ ^]^

The splice variant rs72613567T>TA in *HSD17B13* has been consistently associated with a lower grade of lobular inflammation, NASH, and stage of fibrosis in adults,^[^ ^10^ ^]^ although without any difference in severity of histologic steatosis.^[^ ^16^, ^33^ ^]^ In children, we observed a strong negative association with grade of periportal inflammation with no effect on lobular inflammation. Pediatric NASH is often characterized by a “zone 1” predominant distribution of steatosis and inflammation, particularly in younger children.^[^ ^26^ ^]^ While it has been speculated that the periportal inflammation of pediatric NASH “transitions” to lobular inflammation (and ballooning) of adult NASH, it is challenging to prove. Here, we have observed a genetic variant that has a specific association with periportal inflammation in children and with lobular inflammation in adults. We believe this provides further evidence to support the notion that pediatric NASH shares similar genetics to adult NASH, despite having a different histologic pattern.

Several groups have previously demonstrated that the splice variant rs72613567T>TA in *HSD17B13* is associated with reduced expression of the enzyme,^[^ ^10^, ^16^, ^33^ ^]^ which we have replicated using proteomics. Our pathway analysis also suggested an increase in nonsense‐mediated decay, similar to the expression profiling data from Sookoian and Pirola.^[^ ^33^ ^]^ The function of this enzyme is not exactly clear but has been recently implicated in phospholipid metabolism.^[^ ^16^ ^]^ Our proteomic and lipidomic data are generally concordant with reduced expression of HSD17B13 causing perturbation of glycerophospholipid metabolism. We observed a strong positive association with TG chain length and saturation, while the opposite trend was seen for rs738409C>G in *PNPLA3*. This observation is consistent with results from Luukkonen et al.^[^ ^59^ ^]^ who found that this harmful *PNPLA3* variant leads to hepatic retention of very long‐chain polyunsaturated TG such that they are relatively deficient in the serum. *HSD17B13* and *PNPLA3* have both been shown to localize to lipid droplets,^[^ ^9^, ^17^ ^]^ but it is not yet understood to what extent the alteration in TG composition is a primary contributor to the severity of liver disease. It is also interesting to reflect that we did not find the variant in *MTARC1*, a mitochondrial protein not known to bind to lipid droplets, to have any effect on the serum lipid profile, which could indicate a different mechanism of action.

The *MTARC1* variant discovered by Emdin et al.^[^ ^11^ ^]^ was associated with lower odds of all‐cause cirrhosis, diagnosis of fatty liver, and lower liver fat on computed tomography. We did not observe any effect of rs2642438G>A in the *MTARC1* genotype on odds of NAFLD in children. However, in children with established NAFLD severe enough to warrant liver biopsy, we found a lower grade of histologic steatosis and proteomic signatures of reduced fibrogenesis. Our data, therefore, suggest that this variant in MTARC1 is a risk factor for severity but not development of NAFLD in children.

The precise role of MTARC1 in hepatic metabolism is unknown. We found this enzyme to be expressed at similar levels across rs2642438G>A genotypes even though the variant (p.Ala165Thr) would be predicted to have a destabilizing effect. MTARC1 is a molybdenum‐dependent enzyme that reduces N‐oxygenated molecules.^[^ ^19^, ^49^ ^]^ We found that alanine‐165 is highly conserved and threonine‐165 may disrupt the alpha‐helix and its ability to bind molybdenum, although precisely how this reduces the severity of NAFLD will require further characterization.

Our proteomics showed a consistent trend of increased retinol metabolism associated with *HSD17B13* and *MTARC1* variants, while it was reduced with the *PNPLA3* variant, as reported previously.^[^ ^30^ ^]^ While it is possible that a specific common mechanism underlies this, it may also be a secondary observation. Mechanistic work does implicate *PNPLA3* ^[^ ^60^ ^]^ and *HSD17B13* in retinol metabolism,^[^ ^17^ ^]^ but it is not known for *MTARC1*. These results could also be accounted for by activation of hepatic stellate cells^[^ ^61^ ^]^ in the context of more advanced NAFLD, with an accompanying down‐regulation of their retinol metabolism. More generally, it is unclear whether disordered retinol metabolism is causal in the severity of NAFLD.

The strengths of this study include a comparatively large number of histologically characterized children and use of unbiased lipidomics and proteomics data to give insights into variant function. Also, replication of well‐established associations with rs738409C>G in *PNPLA3* provides further confidence in our findings. Studying pediatric subjects with NAFLD reduces the risk of interaction with factors attributable to adult multimorbidity or substance toxicity.

In this study, we were unable to account for genetic ancestry in analyses due to use of genotyping individual variants. In addition, we may have had reduced power for case–control analyses by use of ultrasound for exclusion of steatosis rather than more sensitive techniques. Therefore, some children with mild steatosis may have been assigned to the control group. Finally, comparatively few children with advanced NAFLD (i.e., cirrhosis) were included, which may have reduced the power to identify further histologic associations.

Future mechanistic work to understand the *MTARC1* variant could focus on the localization of the mutant forms, measurement of its enzymatic activity, and expression with *in vitro* systems to observe interaction with lipid droplets. More broadly, there remains an ongoing need for larger, unbiased, genome‐wide studies of fatty liver disease in children to identify whether novel variants play a role outside of those implicated in adult liver disease.

rs72613567T>TA in *HSD17B13* and rs2642438G>A in *MTARC1* are protective against severity of pediatric NAFLD, suggesting shared genetic influences between adults and children. The two variants have distinct mechanisms: the variant in *HSD17B13* reduces hepatic HSD17B13 expression and perturbs TG and phospholipid metabolism, whereas the *MTARC1* variant had no effect on hepatic MTARC1 expression or lipid metabolism; however, modeling shows that p.Ala165Thr is destabilizing and reduces the metal‐binding capacity MTARC1.

## CONFLICT OF INTEREST

The authors have no conflict of interest to declare.

## AUTHOR CONTRIBUTIONS

*Study concept and design*: Christian A. Hudert, Jake P. Mann, Bart G. Koot, Anita Vreugdenhil, Anna Alisi. *Acquisition of data*: Christian A. Hudert, Leon A. Adams, Anna Alisi, Annalisa Crudele, Laura G. Draijer, Samuel Furse, Benjamin Jenkins, Kylie Karnebeek, Deirdre A. Kelly, Bart G. Koot, Trevor A. Mori, Stuart G. Snowden, Indra van Mourik, Anita Vreugdenhil, Jan G. Hengstler, David Meierhofer, Jake P. Mann. *Analysis and interpretation of data*: Christian A. Hudert, Jake P. Mann, Bart G. Koot, Anita Vreugdenhil, Anna Alisi, David Meierhofer, Quentin M. Anstee. *Drafting of the manuscript*: Christian A. Hudert, Jake P. Mann. *Critical revision of the manuscript for important intellectual content*: Christian A. Hudert, Leon A. Adams, Anna Alisi, Annalisa Crudele, Laura G. Draijer, Samuel Furse, Benjamin Jenkins, Kylie Karnebeek, Deirdre A. Kelly, Albert Koulman, Bart G. Koot, Trevor A. Mori, Stuart G. Snowden, Indra van Mourik, Anita Vreugdenhil, Jan G. Hengstler, David Meierhofer, Jake P. Mann, Quentin M. Anstee. *Statistical analysis*: Christian A. Hudert, Samuel Furse, Stuart G. Snowden, David Meierhofer, Jake P. Mann. *Obtained funding*: Christian A. Hudert, Bart G. Koot, Trevor A. Mori, Anita Vreugdenhil, Anna Alisi, Albert Koulman, Jake P. Mann. *Administrative, technical, or material support*: Annalisa Crudele, Kylie Karnebeek, Benjamin Jenkins. *Study supervision*: Christian A. Hudert, Jake P. Mann.

## PREPRINT

This manuscript is available as a preprint on medRxiv: https://www.medrxiv.org/content/10.1101/2020.06.05.20120956v1

## Supporting information

Supplementary Material

Table S1‐S7, S10‐S11

Table S8‐S9, S12‐S13

## Data Availability

Summary statistics from all analyses are available in [Link], [Link]. Further details are available from the corresponding author on request.
