# Supplementary material for: Variants in mitochondrial amidoxime reducing component 1 and hydroxysteroid 17‐beta dehydrogenase 13 reduce severity of nonalcoholic fatty liver disease in children and suppress fibrotic pathways through distinct mechanisms
Source: Hepatol Commun. 2022 Apr 11;6(8):1934–48. doi: 10.1002/hep4.1955 (PMC9315139; doi:10.1002/hep4.1955)
Supplement: Supplementary file 1 — Supplementary Material [file HEP4-6-1934-s002.pdf]

Variants in *MARC1* and *HSD17B13* reduce severity of NAFLD in children, perturb phospholipids, and suppress fibrotic pathways

Supplementary Methods 2
Hepatic proteomic analysis 2
Plasma lipidomics analysis 4
Supplementary Figures 6
Supplementary Figure 1. 6
Supplementary Figure 2. 7
Supplementary Tables 8
Supplementary Table 1. 8
Supplementary Table 2. 9
Supplementary Table 3. 10
Supplementary Table 4. 11
Supplementary Table 5. 12
Supplementary Table 6. 14
Supplementary Table 7. 15
Supplementary Table 8 [Excel spreadsheet]. 16
Supplementary Table 9 [Excel spreadsheet]. 16
Supplementary Table 10. 17
Supplementary Table 11. 19
Supplementary Table 12 [Excel spreadsheet]. 20
Supplementary Table 13 [Excel spreadsheet]. 20

Supplementary Methods

Hepatic proteomic analysis

A subset of 70 liver tissue specimens were homogenized under denaturing conditions with a FastPrep in a buffer containing 3 M GdmCL, 5 mM TCEP, 20 mM CAA, and 50 mM Tris pH 8.5, followed by sonication for 15 min and boiled at 95 °C for 15 min. The lysates were digested and purified using the preOmics in-stage tip kit (iST kit 96x, Martinsried, Germany). Samples were eluted sequentially in three fractions using the SDB-RPS-1 and -2 buffers[1] and the elution buffer provided by preOmics for subsequent analysis on a nano LC-MS/MS.

LC−MS/MS was carried out by nanoflow reverse-phase liquid chromatography (Dionex Ultimate 3000, Thermo Scientific, Waltham, MA) coupled online to a Q-Exactive HF Orbitrap mass spectrometer (Thermo Scientific, Waltham, MA). The LC separation was performed using a PicoFrit analytical column (75 μm ID × 55 cm long, 15 µm Tip ID (New Objectives, Woburn, MA) packed in-house with 3-µm C18 resin (Reprosil-AQ Pur, Dr. Maisch, Ammerbuch-Entringen, Germany), as reported previously (Gielisch and Meierhofer 2015). Peptides were eluted using a gradient from 3.8 to 50 % solvent B in solvent A over 121 min at 266 nL per minute flow rate. Solvent A was 0.1 % formic acid and solvent B was 79.9 % acetonitrile, 20 % H_2O, 0.1 % formic acid. Nanoelectrospray was generated by applying 3.5 kV. A cycle of one full Fourier transformation scan mass spectrum (300−1750 m/z, resolution of 60,000 at m/z 200, AGC target $1e^6$) was followed by 12 data-dependent MS/MS scans scans (resolution of 30,000, AGC target $5e^5$) with a normalized collision energy of 25 eV. In order to avoid repeated sequencing of the same peptides, a dynamic exclusion window of 30 seconds was used. Additionally, only peptide charge states between two and eight were sequenced.

Raw MS data were processed with MaxQuant software (v1.6.0.1)[2] and searched against the human proteome database UniProtKB with 70,941 entries, released in 01/2017. Parameters of MaxQuant database searching were as follows: a false discovery rate (FDR) of 0.01 for proteins and peptides, a minimum peptide length of 7 amino acids, a mass tolerance of 4.5 ppm for precursor and 20 ppm

for fragment ions. A maximum of two missed cleavages was allowed for the tryptic digest. Cysteine carbamidomethylation was set as fixed modification, while N-terminal acetylation and methionine oxidation were set as variable modifications.
Proteomics data were logarithmically transformed and then standardised to mean = 0 and standard deviation = 1. Only proteins with detectable abundance of >70% in each group were included in analyses, imputation from normal distribution was used for missing (or zero) values. Association between individual proteins and genotype was assessed according to an additive genetic model using linear regression, with proteins as dependent variable and genotype (coded as 0, 1, or 2 effect alleles), age, and sex and independent variables. Beta regression coefficient therefore represents the change in log-standard deviations of protein abundance per effect allele, adjusted for age and sex.

In addition, a two-sample t-test was performed comparing homozygous wild-type against homozygous mutants (e.g. rs72613567 T/T vs. TA/TA. Multiple test correction was done by Benjamini-Hochberg with an FDR of 0.05 by using Perseus (v1.6.0.2)[3].

For comprehensive proteome data analyses, gene set enrichment analysis (GSEA, v4.0.0)[4] was applied in order to see if *a priori* defined sets of proteins show statistically significant, concordant differences according to genotype. GSEA was performed using the preranked gene list derived from linear regression analysis. GSEA default settings were used. As recommended for hypothesis discovery, cut off for significantly regulated pathways was set to a *P*-value < 0.05 and an FDR Q-value < 0.25. GSEA results were then passed to Cytoscape[5,6] for plotting of enrichment maps by clustering similar gene sets, using Enrichment Map[7]. Groups of gene sets were annotated using AutoAnnotate[8] with WordCloud[9].

Volcano blot was performed using an FDR Q-value of 0.05 and an artificial within groups variance (s0) of 0.1.

Plasma lipidomics analysis

For lipid profiling, the plasma samples were analyzed by liquid chromatography with mass spectrometry detection (LC–MS) as described previously[10].

All solvents and additives were of HPLC grade or higher and purchased from Sigma Aldrich (Haverhill, Suffolk, UK) unless otherwise stated.

The protein-precipitation liquid extraction protocol has been described previously[10]. Briefly, 50 µL of plasma was transferred into a 2 mL screw cap Eppendorf plastic tube (Eppendorf, Stevenage, UK). Immediately, 650 µL of chloroform was added to each sample, followed by thorough mixing. Then, 100 µL of the LIPID-IS (5 µM in methanol), 100 µL of the CARNITINE-IS (5 µM in methanol) and 150 µL of methanol was added to each sample, followed by thorough mixing. Then, 400 µL of acetone was added to each sample. The samples were vortexed and centrifuged for 10 minutes at ~20,000 g to pellet any insoluble material. The supernatant was pipetted into separate 2 mL screw cap amber-glass auto-sampler vials (Agilent Technologies, Cheadle, United Kingdom). The organic extracts were dried down to dryness using a Concentrator Plus system (Eppendorf, Stevenage, UK) run for 60 minutes at 60 degree Celsius. The samples were reconstituted in 100 µL of 2: 1: 1 (propan-2-ol, acetonitrile and water, respectively) then thoroughly vortex. The reconstituted sample was transferred into a 250 µL low-volume vial insert inside a 2 mL amber glass auto-sample vial ready for liquid chromatography with mass spectrometry detection (LC-MS) analysis.

Full chromatographic separation of intact lipids was achieved using Shimadzu HPLC System (Shimadzu UK Limited, Milton Keynes, United Kingdom) with the injection of 10 µL onto a Waters Acquity UPLC® CSH C18 column (Waters, Hertfordshire, United Kingdom); 1.7 µm, I.D. 2.1 mm X 50 mm, maintained at 55 degrees Celsius. Mobile phase A was 6:4, acetonitrile and water with 10 mM ammonium

formate. Mobile phase B was 9:1, propan-2-ol and acetonitrile with 10 mM ammonium formate. The flow was maintained at 500 µL per minute through the following gradient: 0.00 minutes_40% mobile phase B; 0.40 minutes_43% mobile phase B; 0.45 minutes_50% mobile phase B; 2.40 minutes_54% mobile phase B; 2.45 minutes_70% mobile phase B; 7.00 minutes_99% mobile phase B; 8.00 minutes_99% mobile phase B; 8.3 minutes_40% mobile phase B; 10 minutes_40% mobile phase B. The sample injection needle was washed using 9:1, 2-propan-2-ol and acetonitrile. The mass spectrometer used was the Thermo Scientific Exactive Orbitrap with a heated electrospray ionization source (Thermo Fisher Scientific, Hemel Hempstead, UK). The mass spectrometer was calibrated immediately before sample analysis using positive and negative ionization calibration solution (recommended by Thermo Scientific). Additionally, the mass spectrometer scan rate was set at 4 Hz, giving a resolution of 25,000 (at 200 m/z) with a full-scan range of m/z 100 to 1,800 with continuous switching between positive and negative mode.

Data processing—The instrument responses of the analytes were normalized to the relevant internal standard response (producing area ratios), these area ratios corrected the intensity for any extraction and instrument variations. The area ratios were then blank corrected where intensities less than three times the blank samples were set to a 'Not Found' result (i.e., zero concentration). The accepted area ratios were then multiplied by the concentration of the internal standard to give the analyte semi-quantitative concentrations.

Supplementary Figures

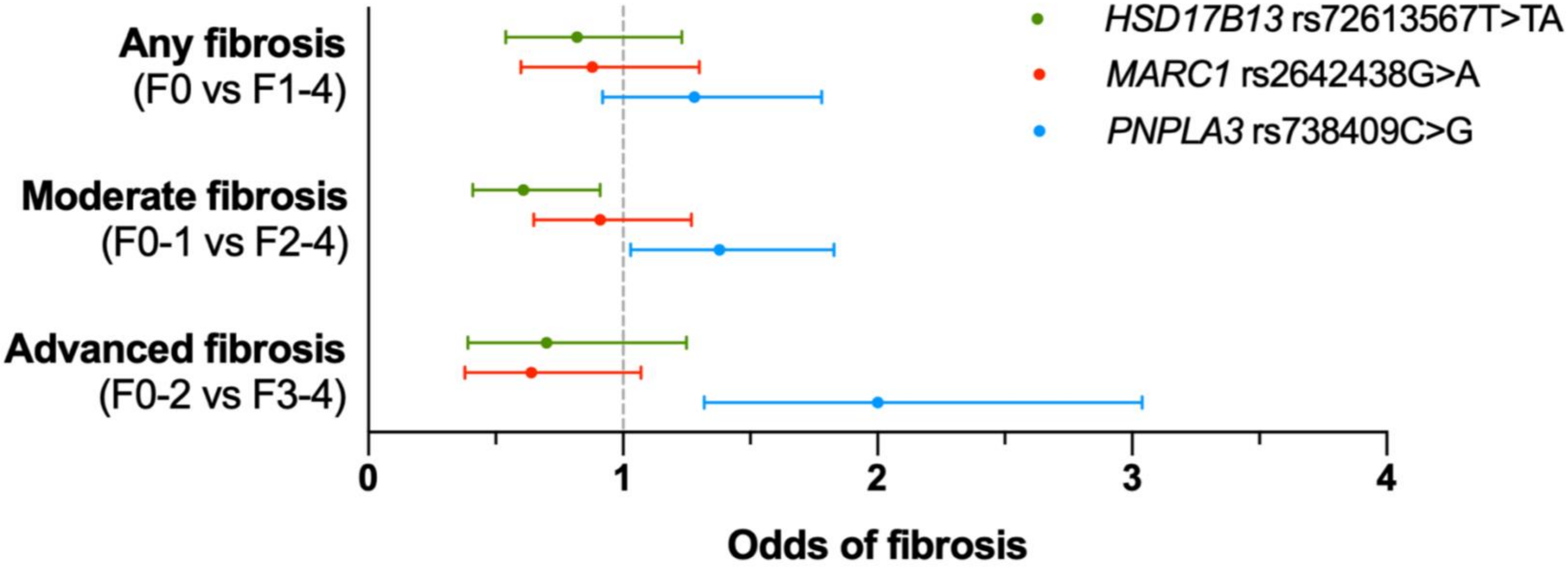

Supplementary Figure 1.
Odds ratios for the presence of any, moderate or advanced fibrosis using an additive genetic model. Data from 406 children with biopsy-proven NAFLD and liver histology. *'PNPLA3'* refers to 738409C>G, *'MARC1'* refers to rs2642438G>A, and *'HSD17B13'* refers to rs72613567T>TA.

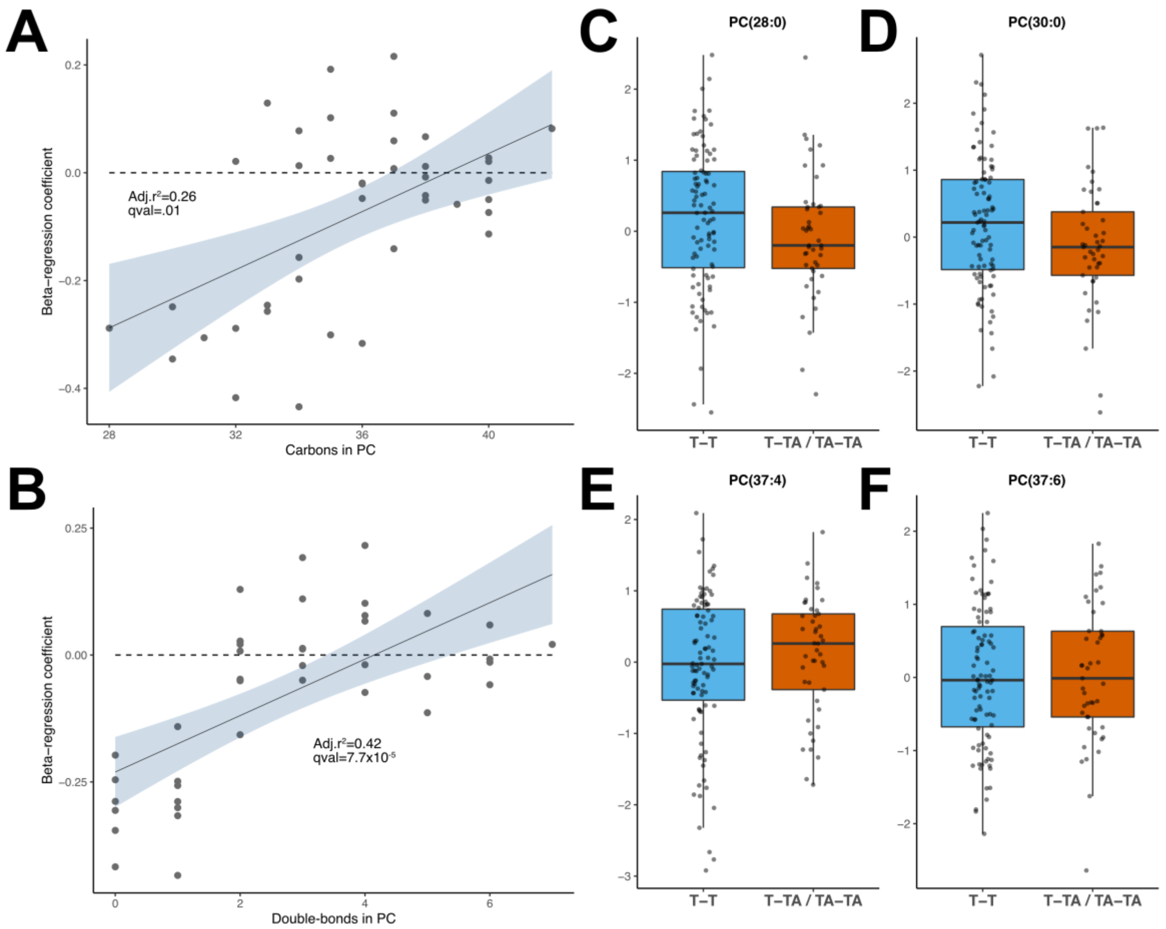

Supplementary Figure 2.
Plasma lipid species associated with rs72613567T>TA in *HSD17B13.* Meta-regression plots demonstrating a positive association with phosphatidylcholine (PC) chain length (A) and saturation (B), where each data point represents a different TG species. C-F, show box plots illustrating differences for individual lipids, where each data point represents a child with NAFLD. Data from 141 children with NAFLD. PC, phosphatidylcholines.

Supplementary Tables

Supplementary Table 1.

SNP	Genotype	Controls	Cases	Genotypic Odds	*P* value	Allelic Odds	*P* value
HSD17B13						0.66 (0.55-0.79)	**<0.0001**
	T/T	358	477	1			
	T/TA	282	219	0.64 (0.50-0.81)	**2.9×10^{-4}**		
	TA/TA	43	33	0.63 (0.37-1.06)	0.080		
	Additive			0.71 (0.58-0.86)	**4.4×10^{-4}**		
	Recessive			0.76 (0.46-1.26)	0.281		
	Dominant			0.64 (0.50-0.80)	**3.3×10^{-8}**		
MTARC1						1.05 (0.44-1.25)	0.599
	GG	362	385	1			
	GA	238	280	1.13 (0.88-1.44)	0.347		
	AA	45	48	1.17 (0.73-1.88)	0.525		
	Additive			1.10 (0.91-1.33)	0.309		
	Recessive			1.11 (0.70-1.77)	0.654		
	Dominant			1.13 (0.90-1.43)	0.296		
PNPLA3						1.83 (1.54-2.19)	**<0.0001**
	CC	332	260	1			
	CG	187	236	1.39 (1.02-1.89)	**0.035**		
	GG	44	106	1.80 (1.15-2.82)	**0.010**		
	Additive			1.32 (1.08-1.63)	**0.008**		
	Recessive			1.44 (0.92-2.24)	0.108		
	Dominant			1.46 (1.10-1.93)	**0.009**		

Note.- *P* values were calculated by binary logistic regression with correction for age, sex and BMI-z for genotype and two-sided Fisher´s exact test for allelic odds ratios.

Supplementary Table 2.

Clinical and laboratory characteristics of all participants stratified to *HSD17B13* genotype.

Variable	T/T (N = 835)	T/TA and TA/TA (N = 577)	*P* value	*Q* value
Age (years)	16.0 (12.6 – 17.0)	16.9 (13.2 – 17.0)	**3.7×10^{-5}**	**1.6×10^{-4}**
Male sex, n (%)	452 (54.1)	300 (52.0)	0.428	0.506
BMI z-score	1.9 (0.8 – 2.7)	1.6 (0.7 – 2.7)	**0.018**	**0.039**
ALT (U/l)	29 (19 – 54)	24 (17 – 40)	**6.0×10^{-6}**	**3.9×10^{-5}**
AST (U/l)	29 (22 – 41)	25 (21 – 33)	**7.0×10^{-7}**	**9.1×10^{-6}**
GGT (U/l)	16 (12 – 26)	16 (12 – 22)	**0.015**	**0.039**
Cholesterol (mg/dl)	158 (139 – 181)	157 (137 – 178)	0.211	0.305
LDL (mg/dl)	93 (77 – 112)	92 (74 – 108)	0.126	0.205
HDL (mg/dl)	45 (39 – 54)	46 (39 – 54)	0.263	0.342
Triglycerides (mg/dl)	89 (67 – 125)	87 (65 – 124)	0.624	0.624
HOMA	2.6 (1.6 – 4.3)	2.4 (1.5 – 3.7)	**0.015**	**0.039**
NAFLD diagnosis, n (%)	477 (43.7)	252 (57.1)	**4.4×10^{-4}**	**0.002**
MTARC1 genotype, n (%)				
GG / GA / AA	443 (55.2)/299 (37.2)/61 (7.6)	304 (54.8)/219 (39.5)/32 (5.8)	0.540	0.585
PNPLA3 genotype, n (%)				
CC / CG / GG	322 (49.1)/231 (35.2)/103 (15.7)	250 (53.1)/174 (36.9)/47 (10.0)	0.117	0.205

Note.- Data represent frequencies (%) or median (interquartile range) as appropriate. For clinical characteristics, *P*-values were calculated using Mann-Whitney U test for continuous traits and Chi-square test for categorical traits. For plasma markers, *P*-values were calculated using linear regression with correction for age and sex. For genotypes, *P*-values were calculated using binary logistic regression with correction for age, sex and BMI-z. FDR correction (*Q* value) for multiple comparisons was calculated using the Benjamini and Hochberg method.

Supplementary Table 3.

Clinical and laboratory characteristics of all participants stratified to *MTARC1* genotype.

Variable	GG (N = 747)	GA and AA (N = 611)	*P* value	*Q* value
Age (years)	16.4 (12.6 – 17.0)	16.1 (12.7 – 17.0)	0.800	0.929
Male sex, n (%)	406 (54.4)	319 (52.2)	0.431	0.929
BMI z-score	1.9 (0.8 – 2.8)	1.8 (0.8 – 2.7)	0.279	0.929
ALT (U/l)	26 (18 – 46)	28 (18 – 49)	0.895	0.929
AST (U/l)	27 (21 – 38)	28 (21 – 40)	0.689	0.929
GGT (U/l)	16 (12– 24)	16 (12 – 25)	0.926	0.929
Cholesterol (mg/dl)	158 (139 – 181)	157 (137 – 179)	0.224	0.929
LDL (mg/dl)	93 (77 – 112)	93 (76 – 109)	0.488	0.929
HDL (mg/dl)	46 (39 – 54)	45 (39 – 53)	0.325	0.929
Triglycerides (mg/dl)	87 (64 – 125)	90 (69 – 124)	0.167	0.929
HOMA	2.5 (1.5 – 4.2)	2.6 (1.6 – 4.0)	0.666	0.929
NAFLD diagnosis, n (%)	385 (53.7)	328 (51.5)	0.599	0.929
HSD17B13 genotype, n (%)				
TT / TTA / TATA	592 (57.7)/368 (35.9)/66 (6.4)	517 (58.7)/309 (35.1)/55 (6.2)	0.913	0.929
PNPLA3 genotype, n (%)				
CC / CG / GG	394 (54.2)/265 (36.5)/68 (9.4)	349 (52.9)/234 (35.5)/77 (11.7)	0.929	0.929

Note.- Data represent frequencies (%) or median (interquartile range) as appropriate. For clinical characteristics, *P*-values were calculated using Mann-Whitney U test for continuous traits and Chi-square test for categorical traits. For plasma markers, *P*-values were calculated using linear regression with correction for age and sex. For genotypes, *P*-values were calculated using binary logistic regression with correction for age, sex and BMI-z. FDR correction (*Q* value) for multiple comparisons was calculated using the Benjamini and Hochberg method.

Supplementary Table 4.

Clinical and laboratory characteristics of patients with liver biopsy.

Variable	NAFLD (N = 729)	NAFLD with liver biopsy (N = 399)
Age (years)	14.0 (12.0 – 16.9)	13.0 (11.0 – 14.6)
Male sex, n (%)	417 (57.2)	243 (60.9)
BMI z-score	2.2 (1.6 – 2.8)	2.1 (1.6 – 2.6)
ALT (U/l)	43 (25 – 73)	53 (31 – 85)
AST (U/l)	34 (24 – 48)	41 (29 – 56)
GGT (U/l)	22 (14 – 33)	23 (15 – 41)
Cholesterol (mg/dl)	160 (139 – 185)	159 (138 – 184)
LDL (mg/dl)	97 (79 – 112)	98 (82 – 112)
HDL (mg/dl)	43 (38 – 50)	43 (38 – 50)
Triglycerides (mg/dl)	98 (70 – 140)	99 (73 – 147)
HOMA	3.4 (2.2 – 5.2)	3.6 (2.5 – 5.4)
HSD17B13 genotype, n (%)		
TT / TTA / TATA	477 (65.4)/219 (30.0)/33 (4.5)	271 (67.9)/113 (28.3)/15 (3.8)
MTARC1 genotype, n (%)		
GG / GA / AA	385 (54.0)/280 (39.3)/48 (6.7)	205 (51.4)/171 (42.9)/23 (5.8)
PNPLA3 genotype, n (%)		
CC / CG / GG	260 (43.2)/236 (39.2)/106 (17.6)	141 (36.6)/162 (42.1)/82 (21.3)

Note.- Data represent frequencies (%) or median (interquartile range) as appropriate. BMI, body mass index; ALT, alanine aminotransferase; AST, aspartate aminotransferase; GGT, gamma glutamyl transferase; LDL, low density lipoprotein ; HDL, high density lipoprotein; HOMA, homeostatic model assessment of insulin resistance .

Supplementary Table 5.

Associations of genetic variants with histologic features of disease severity.

**Histologic Trait**	*HSD17B13*			*MTARC1*			*PNPLA3*		
n (% of genotype)	TT	TTA	TATA	GG	GA	AA	CC	CG	GG
**Steatosis**									
1	57 (21.0)	29 (25.7)	5 (33.3)	38 (18.5)	43 (25.1)	10 (43.5)	39 (27.7)	36 (22.2)	13 (15.9)
2	143 (52.8)	42 (37.2)	1 (6.7)	100 (48.8)	76 (44.4)	10 (43.5)	72 (51.1)	78 (48.1)	32 (39.0)
3	71 (26.2)	42 (37.2)	9 (60.0)	67 (32.7)	52 (30.4)	3 (13.0)	30 (21.3)	48 (29.6)	37 (45.1)
P value, univariate		0.146			**0.014**			**3.66×10^{-4}**	
Multivariate		0.162			**0.016**			**2.80×10^{-4}**	
**Fibrosis**									
0	57 (21.0)	29 (25.9)	4 (26.7)	45 (22.0)	38 (22.4)	7 (30.4)	40 (28.4)	33 (20.4)	16 (19.8)
1	98 (36.2)	50 (44.6)	7 (46.7)	79 (38.5)	68 (40.0)	8 (34.8)	59 (41.8)	63 (38.9)	29 (35.8)
2	77 (28.4)	18 (16.1)	4 (26.7)	48 (23.4)	44 (25.9)	7 (30.4)	32 (22.7)	44 (27.2)	18 (22.2)
3	38 (14.0)	14 (12.5)	0 (0.0)	32 (15.6)	19 (11.2)	1 (4.3)	10 (7.1)	21 (13.0)	17 (21.0)
4	1 (0.4)	1 (0.9)	0 (0.0)	1 (0.5)	1 (0.6)	0 (0.0)	0 (0.0)	1 (0.6)	1 (1.2)
P value, univariate		**0.032**			0.316			**0.004**	
Multivariate		**0.037**			0.312			**0.007**	
**Lobular I.**									
0	52 (19.2)	22 (19.5)	4 (26.7)	38 (18.5)	35 (20.5)	5 (21.7)	33 (23.4)	29 (17.9)	12 (14.6)
1	138 (50.9)	61 (54.0)	7 (46.7)	108 (52.7)	84 (49.1)	14 (60.9)	74 (52.5)	82 (50.6)	42 (51.2)
2	81 (29.9)	30 (26.5)	4 (26.7)	59 (28.8)	52 (30.4)	4 (17.4)	34 (24.1)	51 (31.5)	28 (34.1)
P value, univariate		0.484			0.543			**0.039**	

Multivariate		0.426			0.594			**0.026**	
**Portal I.**									
0	65 (24.4)	43 (39.1)	8 (53.3)	65 (32.3)	45 (26.9)	6 (26.1)	44 (31.2)	51 (32.7)	18 (22.5)
1	167 (62.8)	55 (50.0)	7 (46.7)	108 (53.7)	107 (64.1)	14 (60.9)	83 (58.9)	89 (57.1)	49 (61.3)
2	34 (12.8)	12 (10.9)	0 (0.0)	28 (13.9)	15 (9.0)	3 (13.0)	14 (9.9)	16 (10.3)	13 (16.3)
P value, univariate		**8.96×10^{-4}**			0.666			0.135	
Multivariate		**9.23×10^{-4}**			0.606			0.221	
**Ballooning**									
0	79 (29.6)	39 (35.1)	6 (40.0)	64 (31.5)	52 (31.1)	8 (34.8)	48 (34.0)	50 (31.8)	21 (25.9)
1	116 (43.4)	47 (42.3)	3 (20.0)	84 (41.4)	73 (43.7)	9 (39.1)	61 (43.3)	57 (36.3)	40 (49.4)
2	72 (27.0)	25 (22.5)	6 (40.0)	55 (27.1)	42 (25.1)	6 (26.1)	32 (22.7)	50 (31.8)	20 (24.7)
P value, univariate		0.409			0.776			0.272	
Multivariate		0.375			0.829			0.290	

Note.- Data represent frequencies *P* values were calculated using univariate or multivariate logistic regression with correction for age and sex.

Supplementary Table 6.

Odds ratios for the presence of fibrosis.

SNP	Fibrosis	Odds ratio, additive model	*P* value	Odds ratio, dominant model	*P* value
HSD17B13					
	Any	0.82 (0.54 - 1.23)	0.335	0.77 (0.47 - 1.27)	0.309
	Moderate	0.61 (0.41 - 0.91)	**0.014**	0.56 (0.35 - 0.88)	**0.011**
	Advanced	0.70 (0.39 - 1.25)	0.225	0.77 (0.40 - 1.46)	0.413
MTARC1					
	Any	0.88 (0.60 - 1.30)	0.525	0.92 (0.58 - 1.48)	0.740
	Moderate	0.91 (0.65 - 1.27)	0.575	0.91 (0.61 - 1.36)	0.637
	Advanced	0.64 (0.38 - 1.07)	0.088	0.64 (0.36 - 1.16)	0.141
PNPLA3					
	Any	1.28 (0.92 - 1.78)	0.151	1.51 (0.92 - 2.46)	0.102
	Moderate	1.38 (1.03 - 1.83)	**0.028**	1.67 (1.07 - 2.62)	**0.025**
	Advanced	2.00 (1.32 - 3.04)	**0.001**	2.70 (1.29 - 5.64)	**0.008**

Note.- *P* values were calculated by binary logistic regression with correction for age and sex.

Supplementary Table 7.

Clinical and laboratory characteristics of patients with liver tissue proteomic profiles.

Variable	NAFLD (N = 729)	NAFLD with liver proteomics (N = 70)
Age (years)	14.0 (12.0 – 16.9)	14.0 (13.0 – 16.0)
Male sex, n (%)	417 (57.2)	54 (77.1)
BMI z-score	2.2 (1.6 – 2.8)	2.9 (2.5 – 3.2)
ALT (U/l)	43 (25 – 73)	88 (62 – 125)
AST (U/l)	34 (24 – 48)	52 (39 – 69)
GGT (U/l)	22 (14 – 33)	40 (30 – 56)
Cholesterol (mg/dl)	160 (139 – 185)	163 (146 – 181)
LDL (mg/dl)	97 (79 – 112)	104 (90 – 119)
HDL (mg/dl)	43 (38 – 50)	42 (36 – 51)
Triglycerides (mg/dl)	98 (70 – 140)	118 (93 – 168)
HOMA	3.4 (2.2 – 5.2)	5.9 (4.4 – 9.0)
HSD17B13 genotype, n (%)		
TT / TTA / TATA	477 (65.4)/219 (30.0)/33 (4.5)	50 (71.4)/15 (21.4)/5 (7.1)
MTARC1 genotype, n (%)		
GG / GA / AA	385 (54.0)/280 (39.3)/48 (6.7)	41 (58.6)/23 (32.9)/6 (8.6)
PNPLA3 genotype, n (%)		
CC / CG / GG	260 (43.2)/236 (39.2)/106 (17.6)	20 (29.0)/31 (44.9)/18 (26.1)

Note.- Data represent frequencies (%) or median (interquartile range) as appropriate. For clinical characteristics, *P*-values were calculated using Mann-Whitney U test for continuous traits and Chi-square test for categorical traits. For plasma markers, *P*-values were calculated using linear regression with correction for age and sex. For genotypes, *P*-values were calculated using binary logistic regression with correction for age and sex. FDR correction (*Q* value) for multiple comparisons was calculated using the Benjamini and Hochberg method.

Supplementary Table 8 [Excel spreadsheet].
Hepatic proteomics results for rs72613567T>TA in *HSD17B13* and rs2642438G>A in *MARC1*. Association between proteins and variants was calculated using linear regression (for an additive model coding number of effect alleles as 0, 1, or 2) adjusted for age and sex. Beta regression coefficient therefore represents the change in log-standard deviations of protein abundance per effect allele.

Supplementary Table 9 [Excel spreadsheet].
Gene set enrichment analysis (GSEA) results for rs72613567T>TA in *HSD17B13* and rs2642438G>A in *MARC1* for gene sets with nominal P-value < 0.05 and FDR Q-value <0.25.

Supplementary Table 10.

Predicted consequence of p.Ala165Thr in *MARC1* (from rs2642438G>A) using three *in silico* analysis tools.

Tool	Result for *MARC1* p.Ala165Thr	Explanatory notes
	**Prediction of impact of missense variants on protein function**	
**SNPs&GO**[11,12]	PhD-SNP: Neutral (Prob=0.483, RI=0)<br>PANTHER: Disease (Prob=0.804, RI=6)<br>SNPs&GO: Neutral (Prob=0.455, RI=1)	Several models are integrated to give the overall SNPs&GO output. A probability of >0.5 is predicted as 'Disease'. https://snps.biofold.org/snps-and-go/
**Align-GVGD**[13–15]	GV=0.0<br>GD=58.02<br>Prediction=Class C55	GV is a measure of biochemical variation of the mutation.<br>GD is a measure of the difference in properties of mutation.<br>There are seven classifiers, where C65 is the most likely to interfere with protein function, C55 is the second most likely, and C0 is the least likely. http://agvgd.hci.utah.edu/
**MutPred2**[16]	Score = 0.745<br>Affected motifs:<br>Loss of Helix (Prob=0.27, p=0.05); Altered Metal binding (Prob=0.26, p=9.6e-03); Gain of Relative solvent accessibility (Prob=0.25, p=0.03); Gain of Allosteric site at W168 (Prob=0.21,p=0.03); Gain of Catalytic site at W168 (Prob=0.18, p=0.01); Gain of Disulfide linkage at C161 (Prob=0.15, p=0.03); Loss of Pyrrolidone carboxylic acid at Q167 (Prob=0.10, p=0.01)	Score of >0.5 indicates pathogenicity.<br>MutPred2 also predicts the probability of structural & functional properties and generates a p-value for each to occur compared to the probability of those motifs being altered by benign mutations.<br>A probability of >0.25 is suggested as a threshold for implicating a particular mechanism of pathogenicity, interpreted in combination with the p-value. http://mutpred.mutdb.org/index.html
	**Prediction of impact on protein stability**	
**I-Mutant3.0**[17–19]	ΔΔG Value Prediction: -0.63 kcal/mol<br>SVM2 Prediction Effect: Decrease, RI=7<br>SVM3 Prediction Effect: Large Decrease, RI=3	A negative ΔΔG indicates a decrease in the stability of protein tertiary structure. The tool can either give a binary classification (SMV2) of increase/decrease; or a ternary classification (SMV3) or increase/neutral/decrease. http://gpcr2.biocomp.unibo.it/cgi/predictors/I-Mutant3.0/I-Mutant3.0.cgi
**DUET**[20]	mCSM Predicted Stability Change (ΔΔG):-1.901 kcal/mol (Destabilizing)<br>SDM Predicted Stability Change (ΔΔG):<br>-2.54 Kcal/mol (Destabilizing)<br>DUET Predicted Stability Change (ΔΔG):<br>-2.083 Kcal/mol (Destabilizing)	A tool that combines two previously published approaches (SDM and mCSM) into a single estimate of protein stability, expressed as ΔΔG. This is calculated using the known crystalline structure of MARC1 (6fw2 on PDBe). http://biosig.unimelb.edu.au/duet/stability

**CUPSAT**[21–23]	Overall stability (ΔΔG):-3.74 kcal/mol (Destabilizing), with unfavourable torsion	A tool that combines the physical properties of amino acids with the known crystalline structure of MARC1 (6fw2 on PDBe) to predict the impact on protein stability. http://cupsat.tu-bs.de/

Note.- GD, Grantham Difference; GV, Grantham Variation; mCSM, mutation Cutoff Scanning Matrix; PDBe, Protein Data Bank in Europe; Prob, probability; RI, reliability index; SDM, Site Directed Mutator.

Supplementary Table 11.

Clinical and laboratory characteristics of patients with plasma lipidomics.

Variable	NAFLD (N = 729)	NAFLD with plasma lipidomics (N = 129)
Age (years)	14.0 (12.0 – 16.9)	12.4 (10.4 – 13.4)
Male sex, n (%)	417 (57.2)	67 (52.0)
BMI z-score	2.2 (1.6 – 2.8)	2.08 (1.8 – 2.6)
ALT (U/l)	43 (25 – 73)	60 (41 – 80)
AST (U/l)	34 (24 – 48)	44 (33 – 56)
Cholesterol (mg/dl)	160 (139 – 185)	157 (140 – 190)
LDL (mg/dl)	97 (79 – 112)	99 (90 – 105)
HDL (mg/dl)	43 (38 – 50)	43 (38 – 47)
Triglycerides (mg/dl)	98 (70 – 140)	112 (82 – 155)
HOMA	3.4 (2.2 – 5.2)	3.0 (2.1 – 4.1)
HSD17B13 genotype, n (%)		
TT / TTA / TATA	477 (65.4)/219 (30.0)/33 (4.5)	87 (67.4)/39 (30.2)/3 (2.3)
MTARC1 genotype, n (%)		
GG / GA / AA	385 (54.0)/280 (39.3)/48 (6.7)	62 (48.1)/60 (46.5)/7 (5.4)
PNPLA3 genotype, n (%)		
CC / CG / GG	260 (43.2)/236 (39.2)/106 (17.6)	47 (36.4)/63 (48.8)/19 (14.7)

Note.- Data represent frequencies (%) or median (interquartile range) as appropriate. BMI, body mass index; ALT, alanine aminotransferase; AST, aspartate aminotransferase; LDL, low density lipoprotein ; HDL, high density lipoprotein; HOMA, homeostatic model assessment of insulin resistance .

Supplementary Table 12 [Excel spreadsheet].
Summary statistics for plasma lipidomics for rs72613567T>TA in *HSD17B13*, rs2642438G>A in *MARC1* and s738409C>G in *PNPLA3*. Association between lipids and variants was calculated using logistic regression (wild-type vs. heterozygotes/homozygotes) adjusted for age and sex. Beta regression coefficient therefore represents the change in log-standard deviations of lipid abundance per effect allele. Statistical significance was calculated as $0.05/\sqrt{n}$, due to correlation between lipids, therefore $p<3.3\times10^{-3}$. Data from 141 children with NAFLD.

Supplementary Table 13 [Excel spreadsheet].
Summary statistics for meta-regression analyses from plasma lipidomics for rs72613567T>TA in *HSD17B13*, rs2642438G>A in *MARC1* and s738409C>G in *PNPLA3*. Associations between beta regression co-efficients (from Supplementary Table 12) and lipid chain length (‘Carb’) or saturation (‘db’) using linear regression. All p-values were adjusted for multiple testing using Benjamini-Hochberg method.

Supplementary References

1. Kulak NA, Pichler G, Paron I, et al. Minimal, encapsulated proteomic-sample processing applied to copy-number estimation in eukaryotic cells. Nat Methods 2014;11:319–324.

2. Cox J, Mann M. MaxQuant enables high peptide identification rates, individualized p.p.b.-range mass accuracies and proteome-wide protein quantification. Nat Biotechnol 2008;26:1367–1372.

3. Tyanova S, Temu T, Sinitcyn P, et al. The Perseus computational platform for comprehensive analysis of (prote)omics data. Nat Methods 2016;13:731–740.

4. Subramanian A, Tamayo P, Mootha VK, et al. Gene set enrichment analysis: a knowledge-based approach for interpreting genome-wide expression profiles. Proc Natl Acad Sci U S A 2005;102:15545–15550.

5. Shannon P, Markiel A, Ozier O, et al. Cytoscape: a software environment for integrated models of biomolecular interaction networks. Genome Res 2003;13:2498–2504.

6. Smoot ME, Ono K, Ruscheinski J, et al. Cytoscape 2.8: new features for data integration and network visualization. Bioinformatics 2011;27:431–432.

7. Merico D, Isserlin R, Stueker O, et al. Enrichment map: a network-based method for gene-set enrichment visualization and interpretation. PLoS One 2010;5:e13984.

8. Kucera M, Isserlin R, Arkhangorodsky A, et al. AutoAnnotate: A Cytoscape app for summarizing networks with semantic annotations. F1000Res 2016;5:1717.

9. Oesper L, Merico D, Isserlin R, et al. WordCloud: a Cytoscape plugin to create a visual semantic summary of networks. Source Code Biol Med 2011;6:7.

10. Jenkins B, Ronis M, Koulman A. LC-MS Lipidomics: Exploiting a Simple High-Throughput Method for the Comprehensive Extraction of Lipids in a Ruminant Fat Dose-Response Study. Metabolites 2020;10. Available at: http://dx.doi.org/10.3390/metabo10070296.

11. Capriotti E, Calabrese R, Fariselli P, et al. WS-SNPs&GO: a web server for predicting the deleterious effect of human protein variants using functional annotation. BMC Genomics 2013;14 Suppl 3:S6.

12. Calabrese R, Capriotti E, Fariselli P, et al. Functional annotations improve the predictive score of human disease-related mutations in proteins. Hum Mutat 2009;30:1237–1244.

13. Tavtigian SV, Deffenbaugh AM, Yin L, et al. Comprehensive statistical study of 452 BRCA1 missense substitutions with classification of eight recurrent substitutions as neutral. J Med Genet 2006;43:295–305.

14. Tavtigian SV, Byrnes GB, Goldgar DE, et al. Classification of rare missense substitutions, using risk surfaces, with genetic- and molecular-epidemiology applications. Hum Mutat 2008;29:1342–1354.

15. Mathe E, Olivier M, Kato S, et al. Computational approaches for predicting the biological effect of p53 missense mutations: a comparison of three sequence analysis based methods. Nucleic Acids Res 2006;34:1317–1325.

16. Pejaver V, Urresti J, Lugo-Martinez J, et al. MutPred2: inferring the molecular and phenotypic impact of amino acid variants. bioRxiv 2017:134981. Available at: https://www.biorxiv.org/content/10.1101/134981v1 [Accessed July 25, 2020].

17. Capriotti E, Fariselli P, Casadio R. I-Mutant2.0: predicting stability changes upon mutation from the protein sequence or structure. Nucleic Acids Res 2005;33:W306-10.

18. Capriotti E, Calabrese R, Casadio R. Predicting the insurgence of human genetic diseases associated to single point protein mutations with support vector machines and evolutionary information. Bioinformatics 2006;22:2729–2734.

19. Capriotti E, Fariselli P, Calabrese R, et al. Predicting protein stability changes from sequences using support vector machines. Bioinformatics 2005;21 Suppl 2:ii54-8.

20. Pires DEV, Ascher DB, Blundell TL. DUET: a server for predicting effects of mutations on protein stability using an integrated computational approach. Nucleic Acids Res 2014;42:W314-9.

21. Parthiban V, Gromiha MM, Schomburg D. CUPSAT: prediction of protein stability upon point mutations. Nucleic Acids Res 2006;34:W239-42.

22. Parthiban V, Gromiha MM, Hoppe C, et al. Structural analysis and prediction of protein mutant stability using distance and torsion potentials: role of secondary structure and solvent accessibility. Proteins 2007;66:41–52.

23. Parthiban V, Gromiha MM, Abhinandan M, et al. Computational modeling of protein mutant stability: analysis and optimization of statistical potentials and structural features reveal insights into prediction model development. BMC Struct Biol 2007;7:54.
