# Supplementary material for: Variants in mitochondrial amidoxime reducing component 1 and hydroxysteroid 17‐beta dehydrogenase 13 reduce severity of nonalcoholic fatty liver disease in children and suppress fibrotic pathways through distinct mechanisms
Source: Hepatol Commun. 2022 Apr 11;6(8):1934–48. doi: 10.1002/hep4.1955 (PMC9315139; doi:10.1002/hep4.1955)
Supplement: Supplementary file 2 — Table S1‐S7, S10‐S11 [file HEP4-6-1934-s001.docx]

| **Supplementary Table 1.**  **Odds ratios for the presence of NAFLD.** | | | | | | | | | |  |
| --- | --- | --- | --- | --- | --- | --- | --- | --- | --- | --- |
| SNP | Genotype | Controls | Cases | Genotypic Odds | *P* value | Allelic Odds | | *P* value |  |  |
| *HSD17B13* |  |  |  |  |  | | 0.66 (0.55-0.79) | **<0.0001** |  |  |
|  | T/T | 358 | 477 | 1 |  | |  |  |  |  |
|  | T/TA | 282 | 219 | 0.64 (0.50-0.81) | **2.9 x 10^-4^** | |  |  |  |  |
|  | TA/TA | 43 | 33 | 0.63 (0.37-1.06) | 0.080 | |  |  |  |  |
|  | Additive |  |  | 0.71 (0.58-0.86) | **4.4 x 10^-4^** | |  |  |  |  |
|  | Recessive |  |  | 0.76 (0.46-1.26) | 0.281 | |  |  |  |  |
|  | Dominant |  |  | 0.64 (0.50-0.80) | **3.3 x 10^-8^** | |  |  |  |  |
| *MTARC1* |  |  |  |  |  | | 1.05 (0.44-1.25) | 0.599 |  |  |
|  | GG | 362 | 385 | 1 |  | |  |  |  |  |
|  | GA | 238 | 280 | 1.13 (0.88-1.44) | 0.347 | |  |  |  |  |
|  | AA | 45 | 48 | 1.17 (0.73-1.88) | 0.525 | |  |  |  |  |
|  | Additive |  |  | 1.10 (0.91-1.33) | 0.309 | |  |  |  |  |
|  | Recessive |  |  | 1.11 (0.70-1.77) | 0.654 | |  |  |  |  |
|  | Dominant |  |  | 1.13 (0.90-1.43) | 0.296 | |  |  |  |  |
| *PNPLA3* |  |  |  |  |  | | 1.83 (1.54-2.19) | **<0.0001** |  |  |
|  | CC | 332 | 260 | 1 |  | |  |  |  |  |
|  | CG | 187 | 236 | 1.39 (1.02-1.89) | **0.035** | |  |  |  |  |
|  | GG | 44 | 106 | 1.80 (1.15-2.82) | **0.010** | |  |  |  |  |
|  | Additive |  |  | 1.32 (1.08-1.63) | **0.008** | |  |  |  |  |
|  | Recessive |  |  | 1.44 (0.92-2.24) | 0.108 | |  |  |  |  |
|  | Dominant |  |  | 1.46 (1.10-1.93) | **0.009** | |  |  |  |  |

Note.- *P* values were calculated by binary logistic regression with correction for age, sex and BMI-z for genotype and two-sided Fisher´s exact test for allelic odds ratios.

|  | **Supplementary Table 2.** | | | | | |
| --- | --- | --- | --- | --- | --- | --- |
|  | **Clinical and laboratory characteristics of all participants stratified to *HSD17B13* genotype.** | | | | | |
| Variable | | T/T (N = 835) | T/TA and TA/TA (N = 577) | *P* value | *Q* value |  |
| Age (years) | | 16.0 (12.6 – 17.0) | 16.9 (13.2 – 17.0) | **3.7 x 10^-5^** | **1.6 x 10^-4^** |  |
| Male sex, n (%) | | 452 (54.1) | 300 (52.0) | 0.428 | 0.506 |  |
| BMI z-score | | 1.9 (0.8 – 2.7) | 1.6 (0.7 – 2.7) | **0.018** | **0.039** |  |
| ALT (U/l) | | 29 (19 – 54) | 24 (17 – 40) | **6.0 x 10^-6^** | **3.9 x 10^-5^** |  |
| AST (U/l) | | 29 (22 – 41) | 25 (21 – 33) | **7.0 x 10^-7^** | **9.1 x 10^-6^** |  |
| GGT (U/l) | | 16 (12 – 26) | 16 (12 – 22) | **0.015** | **0.039** |  |
| Cholesterol (mg/dl) | | 158 (139 – 181) | 157 (137 – 178) | 0.211 | 0.305 |  |
| LDL (mg/dl) | | 93 (77 – 112) | 92 (74 – 108) | 0.126 | 0.205 |  |
| HDL (mg/dl) | | 45 (39 – 54) | 46 (39 – 54) | 0.263 | 0.342 |  |
| Triglycerides (mg/dl) | | 89 (67 – 125) | 87 (65 – 124) | 0.624 | 0.624 |  |
| HOMA | | 2.6 (1.6 – 4.3) | 2.4 (1.5 – 3.7) | **0.015** | **0.039** |  |
| NAFLD diagnosis, n (%) | | 477 (43.7) | 252 (57.1) | **4.4 x 10^-4^** | **0.002** |  |
| *MTARC1* genotype, n (%) | |  |  |  |  |  |
| GG / GA / AA | | 443 (55.2)/299 (37.2)/61 (7.6) | 304 (54.8)/219 (39.5)/32 (5.8) | 0.540 | 0.585 |  |
| *PNPLA3* genotype, n (%) | |  |  |  |  |  |
| CC / CG / GG | | 322 (49.1)/231 (35.2)/103 (15.7) | 250 (53.1)/174 (36.9)/47 (10.0) | 0.117 | 0.205 |  |

Note.- Data represent frequencies (%) or median (interquartile range) as appropriate. For clinical characteristics, *P*-values were calculated using Mann-Whitney U test for continuous traits and Chi-square test for categorical traits. For plasma markers, *P*-values were calculated using linear regression with correction for age and sex. For genotypes, *P*-values were calculated using binary logistic regression with correction for age, sex and BMI-z. FDR correction (*Q* value) for multiple comparisons was calculated using the Benjamini and Hochberg method.

|  | **Supplementary Table 3.** | | | | | |
| --- | --- | --- | --- | --- | --- | --- |
|  | **Clinical and laboratory characteristics of all participants stratified to *MTARC1* genotype.** | | | | | |
| Variable | | GG (N = 747) | GA and AA (N = 611) | *P* value | *Q* value |  |
| Age (years) | | 16.4 (12.6 – 17.0) | 16.1 (12.7 – 17.0) | 0.800 | 0.929 |  |
| Male sex, n (%) | | 406 (54.4) | 319 (52.2) | 0.431 | 0.929 |  |
| BMI z-score | | 1.9 (0.8 – 2.8) | 1.8 (0.8 – 2.7) | 0.279 | 0.929 |  |
| ALT (U/l) | | 26 (18 – 46) | 28 (18 – 49) | 0.895 | 0.929 |  |
| AST (U/l) | | 27 (21 – 38) | 28 (21 – 40) | 0.689 | 0.929 |  |
| GGT (U/l) | | 16 (12– 24) | 16 (12 – 25) | 0.926 | 0.929 |  |
| Cholesterol (mg/dl) | | 158 (139 – 181) | 157 (137 – 179) | 0.224 | 0.929 |  |
| LDL (mg/dl) | | 93 (77 – 112) | 93 (76 – 109) | 0.488 | 0.929 |  |
| HDL (mg/dl) | | 46 (39 – 54) | 45 (39 – 53) | 0.325 | 0.929 |  |
| Triglycerides (mg/dl) | | 87 (64 – 125) | 90 (69 – 124) | 0.167 | 0.929 |  |
| HOMA | | 2.5 (1.5 – 4.2) | 2.6 (1.6 – 4.0) | 0.666 | 0.929 |  |
| NAFLD diagnosis, n (%) | | 385 (53.7) | 328 (51.5) | 0.599 | 0.929 |  |
| *HSD17B13* genotype, n (%) | |  |  |  |  |  |
| TT / TTA / TATA | | 592 (57.7)/368 (35.9)/66 (6.4) | 517 (58.7)/309 (35.1)/55 (6.2) | 0.913 | 0.929 |  |
| *PNPLA3* genotype, n (%) | |  |  |  |  |  |
| CC / CG / GG | | 394 (54.2)/265 (36.5)/68 (9.4) | 349 (52.9)/234 (35.5)/77 (11.7) | 0.929 | 0.929 |  |

Note.- Data represent frequencies (%) or median (interquartile range) as appropriate. For clinical characteristics, *P*-values were calculated using Mann-Whitney U test for continuous traits and Chi-square test for categorical traits. For plasma markers, *P*-values were calculated using linear regression with correction for age and sex. For genotypes, *P*-values were calculated using binary logistic regression with correction for age, sex and BMI-z. FDR correction (*Q* value) for multiple comparisons was calculated using the Benjamini and Hochberg method.

|  | **Supplementary Table 4.** | | | | | |
| --- | --- | --- | --- | --- | --- | --- |
|  | **Clinical and laboratory characteristics of patients with liver biopsy.** | | | | | |
| Variable | | NAFLD (N = 729) | | NAFLD with liver biopsy (N = 399) |  |  |
| Age (years) | | 14.0 (12.0 – 16.9) | 13.0 (11.0 – 14.6) | |  |  |
| Male sex, n (%) | | 417 (57.2) | 243 (60.9) | |  |  |
| BMI z-score | | 2.2 (1.6 – 2.8) | 2.1 (1.6 – 2.6) | |  |  |
| ALT (U/l) | | 43 (25 – 73) | 53 (31 – 85) | |  |  |
| AST (U/l) | | 34 (24 – 48) | 41 (29 – 56) | |  |  |
| GGT (U/l) | | 22 (14 – 33) | 23 (15 – 41) | |  |  |
| Cholesterol (mg/dl) | | 160 (139 – 185) | 159 (138 – 184) | |  |  |
| LDL (mg/dl) | | 97 (79 – 112) | 98 (82 – 112) | |  |  |
| HDL (mg/dl) | | 43 (38 – 50) | 43 (38 – 50) | |  |  |
| Triglycerides (mg/dl) | | 98 (70 – 140) | 99 (73 – 147) | |  |  |
| HOMA | | 3.4 (2.2 – 5.2) | 3.6 (2.5 – 5.4) | |  |  |
| *HSD17B13* genotype, n (%) | |  |  | |  |  |
| TT / TTA / TATA | | 477 (65.4)/219 (30.0)/33 (4.5) | 271 (67.9)/113 (28.3)/15 (3.8) | |  |  |
| *MTARC1* genotype, n (%) | |  |  | |  |  |
| GG / GA / AA | | 385 (54.0)/280 (39.3)/48 (6.7) | 205 (51.4)/171 (42.9)/23 (5.8) | |  |  |
| *PNPLA3* genotype, n (%) | |  |  | |  |  |
| CC / CG / GG | | 260 (43.2)/236 (39.2)/106 (17.6) | 141 (36.6)/162 (42.1)/82 (21.3) | |  |  |

Note.- Data represent frequencies (%) or median (interquartile range) as appropriate. BMI, body mass index; ALT, alanine aminotransferase; AST, aspartate aminotransferase; GGT, gamma glutamyl transferase; LDL, low density lipoprotein ; HDL, high density lipoprotein; HOMA, homeostatic model assessment of insulin resistance .

|  | **Supplementary Table 5.** | | | | |
| --- | --- | --- | --- | --- | --- |
|  | **Associations of genetic variants with histologic features of disease severity.** | | | | |
| **Histologic Trait** | | *HSD17B13* | *MTARC1* | *PNPLA3* |  |
| n (% of genotype) | | TT TTA TATA | GG GA AA | CC CG GG |  |
| **Steatosis**  1  2  3 | | 57 (21.0) 29 (25.7) 5 (33.3)  143 (52.8) 42 (37.2) 1 (6.7)  71 (26.2) 42 (37.2) 9 (60.0) | 38 (18.5) 43 (25.1) 10 (43.5)  100 (48.8) 76 (44.4) 10 (43.5)  67 (32.7) 52 (30.4) 3 (13.0) | 39 (27.7) 36 (22.2) 13 (15.9)  72 (51.1) 78 (48.1) 32 (39.0)  30 (21.3) 48 (29.6) 37 (45.1) |  |
| *P* value, univariate | | 0.146 | **0.014** | **3.66 x 10^-4^** |  |
| Multivariate | | 0.162 | **0.016** | **2.80 x 10^-4^** |  |
| **Fibrosis**  0  1  2  3  4 | | 57 (21.0) 29 (25.9) 4 (26.7)  98 (36.2) 50 (44.6) 7 (46.7)  77 (28.4) 18 (16.1) 4 (26.7)  38 (14.0) 14 (12.5) 0 (0.0)  1 (0.4) 1 (0.9) 0 (0.0) | 45 (22.0) 38 (22.4) 7 (30.4)  79 (38.5) 68 (40.0) 8 (34.8)  48 (23.4) 44 (25.9) 7 (30.4)  32 (15.6) 19 (11.2) 1 (4.3)  1 (0.5) 1 (0.6) 0 (0.0) | 40 (28.4) 33 (20.4) 16 (19.8)  59 (41.8) 63 (38.9) 29 (35.8)  32 (22.7) 44 (27.2) 18 (22.2)  10 (7.1) 21 (13.0) 17 (21.0)  0 (0.0) 1 (0.6) 1 (1.2) |  |
| *P* value, univariate | | **0.032** | 0.316 | **0.004** |  |
| Multivariate | | **0.037** | 0.312 | **0.007** |  |
| **Lobular I.**  0  1  2 | | 52 (19.2) 22 (19.5) 4 (26.7)  138 (50.9) 61 (54.0) 7 (46.7)  81 (29.9) 30 (26.5) 4 (26.7) | 38 (18.5) 35 (20.5) 5 (21.7)  108 (52.7) 84 (49.1) 14 (60.9)  59 (28.8) 52 (30.4) 4 (17.4) | 33 (23.4) 29 (17.9) 12 (14.6)  74 (52.5) 82 (50.6) 42 (51.2)  34 (24.1) 51 (31.5) 28 (34.1) |  |
| *P* value, univariate | | 0.484 | 0.543 | **0.039** |  |
| Multivariate | | 0.426 | 0.594 | **0.026** |  |
| **Portal I.**  0  1  2 | | 65 (24.4) 43 (39.1) 8 (53.3)  167 (62.8) 55 (50.0) 7 (46.7)  34 (12.8) 12 (10.9) 0 (0.0) | 65 (32.3) 45 (26.9) 6 (26.1)  108 (53.7) 107 (64.1) 14 (60.9)  28 (13.9) 15 (9.0) 3 (13.0) | 44 (31.2) 51 (32.7) 18 (22.5)  83 (58.9) 89 (57.1) 49 (61.3)  14 (9.9) 16 (10.3) 13 (16.3) |  |
| *P* value, univariate | | **8.96 x 10^-4^** | 0.666 | 0.135 |  |
| Multivariate | | **9.23 x 10^-4^** | 0.606 | 0.221 |  |
| **Ballooning**  0  1  2 | | 79 (29.6) 39 (35.1) 6 (40.0)  116 (43.4) 47 (42.3) 3 (20.0)  72 (27.0) 25 (22.5) 6 (40.0) | 64 (31.5) 52 (31.1) 8 (34.8)  84 (41.4) 73 (43.7) 9 (39.1)  55 (27.1) 42 (25.1) 6 (26.1) | 48 (34.0) 50 (31.8) 21 (25.9)  61 (43.3) 57 (36.3) 40 (49.4)  32 (22.7) 50 (31.8) 20 (24.7) |  |
| *P* value, univariate | | 0.409 | 0.776 | 0.272 |  |
| Multivariate | | 0.375 | 0.829 | 0.290 |  |

Note.- Data represent frequencies *P* values were calculated using univariate or multivariate logistic regression with correction for age and sex.

| **Supplementary Table 6.**  **Odds ratios for the presence of fibrosis.** | | | | | | | |  |
| --- | --- | --- | --- | --- | --- | --- | --- | --- |
| SNP | Fibrosis | Odds ratio,  additive model | *P* value | Odds ratio,  dominant model | | *P* value |  |  |
| *HSD17B13* |  |  |  | |  |  |  |  |
|  | Any | 0.82 (0.54 - 1.23) | 0.335 | | 0.77 (0.47 - 1.27) | 0.309 |  |  |
|  | Moderate | 0.61 (0.41 - 0.91) | **0.014** | | 0.56 (0.35 - 0.88) | **0.011** |  |  |
|  | Advanced | 0.70 (0.39 - 1.25) | 0.225 | | 0.77 (0.40 - 1.46) | 0.413 |  |  |
| *MTARC1* |  |  |  | |  |  |  |  |
|  | Any | 0.88 (0.60 - 1.30) | 0.525 | | 0.92 (0.58 - 1.48) | 0.740 |  |  |
|  | Moderate | 0.91 (0.65 - 1.27) | 0.575 | | 0.91 (0.61 - 1.36) | 0.637 |  |  |
|  | Advanced | 0.64 (0.38 - 1.07) | 0.088 | | 0.64 (0.36 - 1.16) | 0.141 |  |  |
| *PNPLA3* |  |  |  | |  |  |  |  |
|  | Any | 1.28 (0.92 - 1.78) | 0.151 | | 1.51 (0.92 - 2.46) | 0.102 |  |  |
|  | Moderate | 1.38 (1.03 - 1.83) | **0.028** | | 1.67 (1.07 - 2.62) | **0.025** |  |  |
|  | Advanced | 2.00 (1.32 - 3.04) | **0.001** | | 2.70 (1.29 - 5.64) | **0.008** |  |  |

Note.- *P* values were calculated by binary logistic regression with correction for age and sex.

|  | **Supplementary Table 7.** | | | |  | |  |
| --- | --- | --- | --- | --- | --- | --- | --- |
|  | **Clinical and laboratory characteristics of patients with liver tissue proteomic profiles.** | | | |  | |  |
| Variable | | NAFLD (N = 729) | NAFLD with liver proteomics (N = 70) | | |  |  |
| Age (years) | | 14.0 (12.0 – 16.9) | 14.0 (13.0 – 16.0) | | |  |  |
| Male sex, n (%) | | 417 (57.2) | 54 (77.1) | | |  |  |
| BMI z-score | | 2.2 (1.6 – 2.8) | 2.9 (2.5 – 3.2) | | |  |  |
| ALT (U/l) | | 43 (25 – 73) | 88 (62 – 125) | | |  |  |
| AST (U/l) | | 34 (24 – 48) | 52 (39 – 69) | | |  |  |
| GGT (U/l) | | 22 (14 – 33) | 40 (30 – 56) | | |  |  |
| Cholesterol (mg/dl) | | 160 (139 – 185) | 163 (146 – 181) | | |  |  |
| LDL (mg/dl) | | 97 (79 – 112) | 104 (90 – 119) | | |  |  |
| HDL (mg/dl) | | 43 (38 – 50) | 42 (36 – 51) | | |  |  |
| Triglycerides (mg/dl) | | 98 (70 – 140) | 118 (93 – 168) | | |  |  |
| HOMA | | 3.4 (2.2 – 5.2) | 5.9 (4.4 – 9.0) | | |  |  |
| *HSD17B13* genotype, n (%) | |  | |  | | | |
| TT / TTA / TATA | | 477 (65.4)/219 (30.0)/33 (4.5) | 50 (71.4)/15 (21.4)/5 (7.1) | | |  |  |
| *MTARC1* genotype, n (%) | |  |  |  | | | |
| GG / GA / AA | | 385 (54.0)/280 (39.3)/48 (6.7) | 41 (58.6)/23 ( 32.9)/6 (8.6) | | |  |  |
| *PNPLA3* genotype, n (%) | |  |  |  | | | |
| CC / CG / GG | | 260 (43.2)/236 (39.2)/106 (17.6) | 20 (29.0)/31 (44.9)/18 (26.1) | | |  |  |

Note.- Data represent frequencies (%) or median (interquartile range) as appropriate. For clinical characteristics, *P*-values were calculated using Mann-Whitney U test for continuous traits and Chi-square test for categorical traits. For plasma markers, *P*-values were calculated using linear regression with correction for age and sex. For genotypes, *P*-values were calculated using binary logistic regression with correction for age and sex. FDR correction (*Q* value) for multiple comparisons was calculated using the Benjamini and Hochberg method.

**Supplementary Table 10.**

**Predicted consequence of p.Ala165Thr in *MTARC1* (from rs2642438G>A) using three *in silico* analysis tools.**

| Tool | Result for *MARC1* p.Ala165Thr | Explanatory notes |
| --- | --- | --- |
| **Prediction of impact of missense variants on protein function** | | |
| **SNPs&GO**^[10,11](https://paperpile.com/c/bzvkeu/ER2k+MYI3)^ | PhD-SNP: Neutral (Prob=0.483, RI=0)  PANTHER: Disease (Prob=0.804, RI=6)  SNPs&GO: Neutral (Prob=0.455, RI=1) | Several models are integrated to give the overall SNPs&GO output. A probability of >0.5 is predicted as ‘Disease’. <https://snps.biofold.org/snps-and-go/> |
| **Align-GVGD**^[12–14](https://paperpile.com/c/bzvkeu/BIdo+y2LV+OEg5)^ | GV=0.0  GD=58.02  Prediction=Class C55 | GV is a measure of biochemical variation of the mutation.  GD is a measure of the difference in properties of mutation.  There are seven classifiers, where C65 is the most likely to interfere with protein function, C55 is the second most likely, and C0 is the least likely. <http://agvgd.hci.utah.edu/> |
| **MutPred2**^[15](https://paperpile.com/c/bzvkeu/GlyT)^ | Score = 0.745  Affected motifs:  Loss of Helix (Prob=0.27, p=0.05); Altered Metal binding (Prob=0.26, p=9.6e-03); Gain of Relative solvent accessibility (Prob=0.25, p=0.03); Gain of Allosteric site at W168 (Prob=0.21,p=0.03); Gain of Catalytic site at W168    (Prob=0.18, p=0.01); Gain of Disulfide linkage at C161 (Prob=0.15, p=0.03); Loss of Pyrrolidone carboxylic acid at Q167 (Prob=0.10, p=0.01) | Score of >0.5 indicates pathogenicity.  MutPred2 also predicts the probability of structural & functional properties and generates a p-value for each to occur compared to the probability of those motifs being altered by benign mutations.  A probability of >0.25 is suggested as a threshold for implicating a particular mechanism of pathogenicity, interpreted in combination with the p-value. <http://mutpred.mutdb.org/index.html> |
| **Prediction of impact on protein stability** | | |
| **I-Mutant3.0**^[16–18](https://paperpile.com/c/bzvkeu/Mrm7+AUxZ+2jTu)^ | ΔΔG Value Prediction: -0.63 kcal/mol  SVM2 Prediction Effect: Decrease, RI=7  SVM3 Prediction Effect: Large Decrease, RI=3 | A negative ΔΔG indicates a decrease in the stability of protein tertiary structure. The tool can either give a binary classification (SMV2) of increase/decrease; or a ternary classification (SMV3) or increase/neutral/decrease. <http://gpcr2.biocomp.unibo.it/cgi/predictors/I-Mutant3.0/I-Mutant3.0.cgi> |
| **DUET**[^19^](https://paperpile.com/c/bzvkeu/idTz) | mCSM Predicted Stability Change (ΔΔG):-1.901 kcal/mol (Destabilizing)  SDM Predicted Stability Change (ΔΔG):  -2.54 Kcal/mol (Destabilizing)  DUET Predicted Stability Change (ΔΔG):  -2.083 Kcal/mol (Destabilizing) | A tool that combines two previously published approaches (SDM and mCSM) into a single estimate of protein stability, expressed as ΔΔG. This is calculated using the known crystalline structure of MARC1 (6fw2 on PDBe). <http://biosig.unimelb.edu.au/duet/stability> |
| **CUPSAT**[^20–22^](https://paperpile.com/c/bzvkeu/2bPi+e846+UDZN) | Overall stability (ΔΔG):-3.74 kcal/mol (Destabilizing), with unfavourable torsion | A tool that combines the physical properties of amino acids with the known crystalline structure of MARC1 (6fw2 on PDBe) to predict the impact on protein stability. <http://cupsat.tu-bs.de/> |

Note.- GD, Grantham Difference; GV, Grantham Variation; mCSM, mutation Cutoff Scanning Matrix; PDBe, Protein Data Bank in Europe; Prob, probability; RI, reliability index; SDM, Site Directed Mutator;

|  | **Supplementary Table 11.** | | | |
| --- | --- | --- | --- | --- |
|  | **Clinical and laboratory characteristics of patients with plasma lipidomics.** | | | |
| Variable | | NAFLD (N = 729) | NAFLD with plasma lipidomics (N = 129) |  |
| Age (years) | | 14.0 (12.0 – 16.9) | 12.4 (10.4 – 13.4) |  |
| Male sex, n (%) | | 417 (57.2) | 67 (52.0) |  |
| BMI z-score | | 2.2 (1.6 – 2.8) | 2.08 (1.8 – 2.6) |  |
| ALT (U/l) | | 43 (25 – 73) | 60 (41 – 80) |  |
| AST (U/l) | | 34 (24 – 48) | 44 (33 – 56) |  |
| Cholesterol (mg/dl) | | 160 (139 – 185) | 157 (140 – 190) |  |
| LDL (mg/dl) | | 97 (79 – 112) | 99 (90 – 105) |  |
| HDL (mg/dl) | | 43 (38 – 50) | 43 (38 – 47) |  |
| Triglycerides (mg/dl) | | 98 (70 – 140) | 112 (82 – 155) |  |
| HOMA | | 3.4 (2.2 – 5.2) | 3.0 (2.1 – 4.1) |  |
| *HSD17B13* genotype, n (%) | |  |  |  |
| TT / TTA / TATA | | 477 (65.4)/219 (30.0)/33 (4.5) | 87 (67.4)/39 (30.2)/3 (2.3) |  |
| *MTARC1* genotype, n (%) | |  |  |  |
| GG / GA / AA | | 385 (54.0)/280 (39.3)/48 (6.7) | 62 (48.1)/60 (46.5)/7 (5.4) |  |
| *PNPLA3* genotype, n (%) | |  |  |  |
| CC / CG / GG | | 260 (43.2)/236 (39.2)/106 (17.6) | 47 (36.4)/63 (48.8)/19 (14.7) |  |

Note.- Data represent frequencies (%) or median (interquartile range) as appropriate. BMI, body mass index; ALT, alanine aminotransferase; AST, aspartate aminotransferase; LDL, low density lipoprotein ; HDL, high density lipoprotein; HOMA, homeostatic model assessment of insulin resistance .
